# YOLO-based high-throughput phenotyping pipeline for soybean nodulation traits in genomic research

**DOI:** 10.3389/fpls.2026.1816132

**Published:** 2026-04-21

**Authors:** Kuber Shivashakarappa, Niraj Ghimire, Lin Wang, Korsi Dumenyo, Shree Pariyar, Wolfgang Busch, Ali Taheri

**Affiliations:** 1 Department of Agricultural Science and Engineering, College of Agriculture, Tennessee State University, Nashville, TN, United States; 2 Plant Molecular and Cellular Biology Laboratory, Salk Institute for Biological Studies, La Jolla, CA, United States

**Keywords:** deep learning, GWAS, nodulation, object detection, phenotyping, root traits, soybean, YOLO

## Abstract

The symbiotic interaction between soybean (*Glycine max*) and *Bradyrhizobium japonicum* results in the formation of root nodules, specialized organs that house nitrogen-fixing bacteria converting atmospheric N_2_ into plant-accessible ammonia (NH_3_). Accurate quantification of nodule traits is essential for understanding host–microbe interactions and genetic determinants of nodulation. However, traditional manual or semi-quantitative approaches are labor-intensive, subjective, and unsuitable for large-scale studies. Here, we present a high-throughput phenotyping pipeline based on the YOLO deep learning architecture for the automated detection and extraction of soybean root traits. The pipeline quantifies nodule count, dimensions, and spatial distribution, enabling measurement of 24 distinct nodulation-related traits. Using root images from 21-day-old hydroponically grown soybean plants, the model achieved a precision of 0.94, a recall of 0.95, and an F1 score of 0.94 for nodule detection, maintaining accuracy across count ranges. It processes 50 root images in 37 seconds on a single GPU (45 GB memory), representing a ~227-fold improvement in efficiency compared to manual scoring (~2 h 20 min). As proof of concept, we applied this pipeline in a genome-wide association study (GWAS) using the FarmCPU approach and identified 50 significant SNPs associated with multiple nodulation traits, including novel ones. Several candidate genes linked to these loci suggest potential new regulators of nodulation. This YOLO-based phenotyping framework provides a robust, scalable, and reproducible tool for trait discovery and genetic analysis, advancing research in legume genomics and crop improvement. To promote the adoption of this user-friendly nodulation phenotyping pipeline and to support its further development, we have made all essential resources publicly available at: https://github.com/Salk-Harnessing-Plants-Initiative/soybean-nodule-detection.

## 1 Introduction

Soybean [*Glycine max* (L.) Merr.] is one of the most important legume crops and a critical component of global food security, owing to its high oil content (~20%) and protein content (~40%), which make it a valuable protein source for both human consumption and animal feed (Silva and Perrone, 2015). Soybean seeds are also rich in micronutrients such as biotin and phytochemicals, including isoflavones and phenolic compounds, often at concentrations exceeding those found in meat, fruits, and vegetables (Agarwal et al., 2013). To meet the nutrient requirements of the projected global population by 2050, it is estimated that soybean production must increase by approximately 140% relative to the production levels of 2000 (Van et al., 2021).

The high protein content of soybeans necessitates substantial nitrogen input, which is primarily met through biological nitrogen fixation (BNF) facilitated by a symbiotic relationship between soybean plants and *Bradyrhizobium* species. These bacteria colonize specialized root structures known as nodules, where they convert atmospheric nitrogen into ammonia, making it available for plant growth (Muldar et al., 2005). In soils rich in inorganic nitrogen, biological nitrogen fixation contributes approximately 25–50% of the nitrogen content in soybean seeds, whereas in soils with low nitrogen and organic matter, the contribution can reach ~80% (Fujikake, 2003**;** Mastrodomenico and Purcell, 2012). This makes BNF a critical alternative to chemical nitrogen fertilizers, which are expensive and environmentally detrimental (Sutton et al., 2011).

The presence, number, and size of root nodules are fundamental indicators of nodulation efficiency in legumes, particularly in soybean (Chaulagain and Frugoli, 2021). Comparative analyses of nodulating and non-nodulating soybean genotypes have demonstrated up to a sixfold increase in nitrogen content in nodulating genotypes across multiple growth stages, underscoring the importance of nodulation for nitrogen fixation (Kohl et al., 1980). Given the impact of nodulation on overall soybean productivity, detailed assessment of nodule development and the plant–nodule interaction is essential for understanding the genetic and molecular basis of soybean–*Bradyrhizobium*-mediated nitrogen fixation. Such knowledge facilitates the development of improved cultivars with enhanced nitrogen-fixing capacity (Ogura and Busch, 2015).

Recent advances in high-throughput sequencing technologies and tissue-specific phenotyping platforms have enabled genomic approaches to successfully identify genomic regions underlying key traits in soybean, including agronomic characteristics (Fang et al., 2017), seed weight (Patel et al., 2025), biomass accumulation (Wang et al., 2023), and root system architecture (Rathore et al., 2024). Applying similar approaches to nodulation requires accurate phenotyping of traits such as nodule count, size, height, width, and spatial distribution on the root system, combined with comprehensive genomic data from diverse soybean populations (Jubery et al., 2021).

Historically, nodulation phenotyping relied on subjective visual assessments of the entire root system, including lateral and secondary roots (Weaver and Frederick, 1974). Researchers introduced a visual scale (1–10) for quantifying nodules on the taproot, where 1 indicates minimal nodulation and 10 indicates abundant nodulation (Hiltbold et al., 1980). Later, this system was refined by developing a rating scale that considered both taproot and lateral root nodulation, allowing simultaneous assessment of nodule count and size (Fenta et al., 2014). However, these manual approaches are labor-intensive, time-consuming, costly, and impractical for large-scale experiments due to the complexity of nodule number, size variation, and positional arrangement (Barbedo, 2012).

To overcome these challenges, researchers developed an automated image-based root analysis system capable of quantifying nodule number, position, and size (Han et al., 2015). More recently, machine learning–based pipelines have been introduced to detect and classify nodules while simultaneously measuring root system characteristics in soybean grown under semi-controlled environments, significantly reducing manual labor (Chung et al., 2020**;** Jubery et al., 2021). While these approaches effectively estimate nodule count and size, they fall short in capturing comprehensive traits such as spatial extent, distribution variability, nodule size statistics, and vertical position statistics. These additional spatial and distributional traits are critical in genomic studies, as their inclusion alongside conventional count and size traits can improve resolution of genomic study and facilitate the identification of loci regulating complex nodulation phenotypes. Furthermore, the integration of such a pipeline with genomic approaches, such as GWAS and genomic prediction, has not been previously demonstrated. This limitation underscores the need for an automated phenotyping pipeline specifically designed to quantify an expanded set of nodulation traits, extending beyond the scope of current methods that were primarily intended for conventional traits. Advances in image-based phenotyping for plant tissues and stress responses have made deep learning approaches, particularly convolutional neural networks (CNNs), a viable solution for improving trait detection accuracy and throughput (Zhao et al., 2019). Among these, the YOLO (You Only Look Once) framework has emerged as a leading real-time object detection model due to its speed and accuracy (Bommasani et al., 2021). YOLO autonomously learns and extracts salient features during training, enabling simultaneous feature extraction and model optimization to minimize loss functions associated with the phenotyping task (Alif and Hussain, 2024). YOLO-based systems have achieved high accuracy in agricultural trait phenotyping, including disease detection in tomato and kiwifruit, physiological deterioration in tomato, stress detection in citrus, and measurement of internode length in cherries and apples (Mirhaji et al., 2021**;** Qi et al., 2022; Gai et al., 2023; Fan et al., 2022**;** Yao et al., 2021**;** Ayalde et al., 2024). In soybean phenotyping applications, YOLO-based approaches have demonstrated strong performance across several targeted tasks. For instance, YOLOv3 has been applied for real-time pest detection under field conditions, showing reliable performance across varying environments (Tetila et al., 2024). Similarly, improved YOLOv5-based models have been developed for soybean pod-type classification, achieving enhanced detection accuracy and computational efficiency (Li Y et al., 2024). In addition, YOLOv8-based segmentation frameworks have been utilized for extracting embryonic radicle traits during germination, enabling precise morphological analysis (Wu et al., 2024), while YOLOv9-based approaches have been employed for soybean seed defect detection with high recall and mAP values (Liu et al., 2025). Due to the success of YOLO-based models for trait phenotyping, the present study employed YOLO to develop an automated phenotyping pipeline for high-throughput extraction of 24 nodulation traits, including the number of soybean nodules, nodule zones along both the vertical and horizontal axes, standard deviations of nodule size across the root system, nodule area, and nodule distribution along the y-axis, from 2D images of soybean roots grown under hydroponic conditions. The primary objective of developing this phenotyping pipeline was to enable its application in downstream genomic studies of soybean nodulation. To date, only a limited number of GWAS have targeted nodulation traits, and these studies have predominantly relied on manual phenotyping methods. Previous studies have largely relied on manual measurements for nodulation-related GWAS. For instance, biomass-related traits, including nodule dry weight, were examined in one study but no significant loci were detected, likely due to high phenotypic variability (Wang et al., 2023). Another study identified loci associated with carbon allocation and symbiotic nitrogen fixation (SNF) by manually quantifying traits such as nodule number, nodule dry mass, and their mean (Krueger et al., 2024). A nodulation-focused GWAS also manually measured seven nodulation-related traits to identify genomic regions and candidate genes (Fan et al., 2025).

Considering this limitation, it is crucial to use phenotype data generated by the pipeline, which encompasses an expanded set of novel nodulation traits, as it not only reduces phenotyping time and labor but also facilitates the identification of significant genomic regions associated with novel traits, thereby providing deeper insights into their genetic architecture. To validate this capability and demonstrate a downstream application, the pipeline was employed in GWAS for multiple nodulation traits, resulting in the identification of significant genomic regions and candidate genes associated with nodulation.

## 2 Materials and methods

### 2.1 Seed germination and plant growth

**Figure 1:**
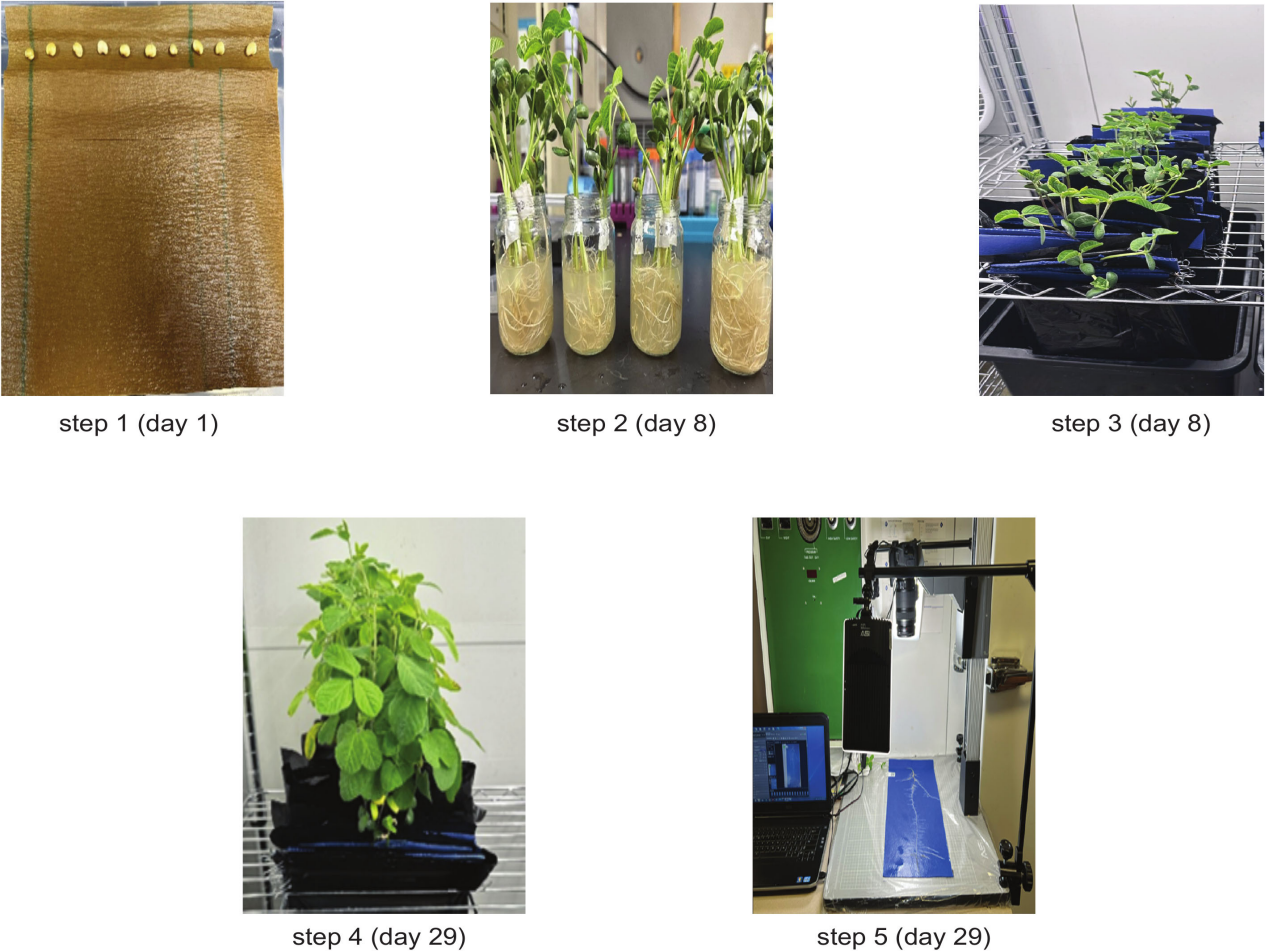
Schematic representation of the experimental workflow: Step 1 (Day 1) – initiation of germination by mounting soybean seeds on brown germination paper; Step 2 (Day 8) – infection of soybean roots with *Bradyrhizobium japonicum*; Step 3 (Day 8) – transfer of seedlings to the hydroponic system; Step 4 (Day 29) – plants prepared for root imaging; Step 5 (Day 29) – capturing of root images using the imaging setup.

A total of 187 soybean genotypes, representing different maturity groups and wide range of geographical origins, were selected from the USDA Germplasm Resources Information Network (GRIN) database (http://www.ars-grin.gov/). The initial step in this study involves growing diverse soybean accessions under hydroponic conditions and capturing root images (**Figure 1**). For that reason, soybean seeds were surface-sterilized in 30% (v/v) bleach with 0.01% Triton X-100 for 15 min, rinsed three times with sterile distilled water, and placed on pre-wetted brown germination paper for uniform germination. The papers were folded, tied, and positioned vertically in containers with a small volume of distilled water, sealed with glass sheets to retain humidity. Seeds germinated for 8 days, with periodic moistening to prevent drying (Langenfeld and Bugbee, 2022). After germination, six uniformly germinated seedlings per accession were carefully unwrapped and immersed in a cell suspension of *Bradyrhizobium japonicum* strain USDA 110 (OD_600_ = 0.8), obtained from the USDA-ARS (https://www.ars.usda.gov/), for 2 hours to allow infection (Nguyen et al., 2012). Following inoculation, seedlings were positioned onto blue blotter paper imprinted with QR code labels encoding accession numbers and replication identifiers. The mounted seedlings were then transferred to a hydroponic system containing a defined nutrient solution. The nutrient solution was prepared with the following macronutrients per liter of final volume: 0.175 g potassium sulfate (K_2_SO_4_), 0.197 g magnesium sulfate heptahydrate (MgSO_4_·7H_2_O), 0.136 g potassium phosphate monobasic (KH_2_PO_4_), and 0.565 g calcium chloride anhydrous (CaCl_2_). An iron source was provided by adding 1.0 mL of a 20 mM Fe-EDTA stock solution per liter. Micronutrients were supplied from a 10× concentrated stock solution, of which 0.5 mL was added per liter of medium. This stock contained 2.86 g/L boric acid (H_3_BO_3_), 1.70 g/L manganese sulfate monohydrate (MnSO_4_·H_2_O), 0.22 g/L zinc sulfate heptahydrate (ZnSO_4_·7H_2_O), 0.08 g/L copper sulfate pentahydrate (CuSO_4_·5H_2_O), and 0.102 g/L molybdic acid (H_3_MoO_4_). The pH of the final solution was adjusted to 6.7 using 2 M KOH. After transfer into the hydroponic system, plants were maintained for 21 days under controlled environmental conditions (16 h light/8 h dark photoperiod, 22–25 °C). During this period, the old nutrient solution was removed and replaced with fresh medium every two days to ensure a consistent supply of mineral nutrients and to support optimal plant development.

After the growth phase, root systems were photographed using a Canon EOS Rebel T5i DSLR camera with a 135 mm lens, controlled by Smart Shooter software for automated, high-resolution image capture. The system used barcode recognition to automatically identify samples and assign filenames from barcoded labels on blue background sheets, eliminating the need for manual input. All images were stored in JPEG format for subsequent analysis.

### 2.2 Soybean nodule detection model development and traits extraction

**Figure 2:**
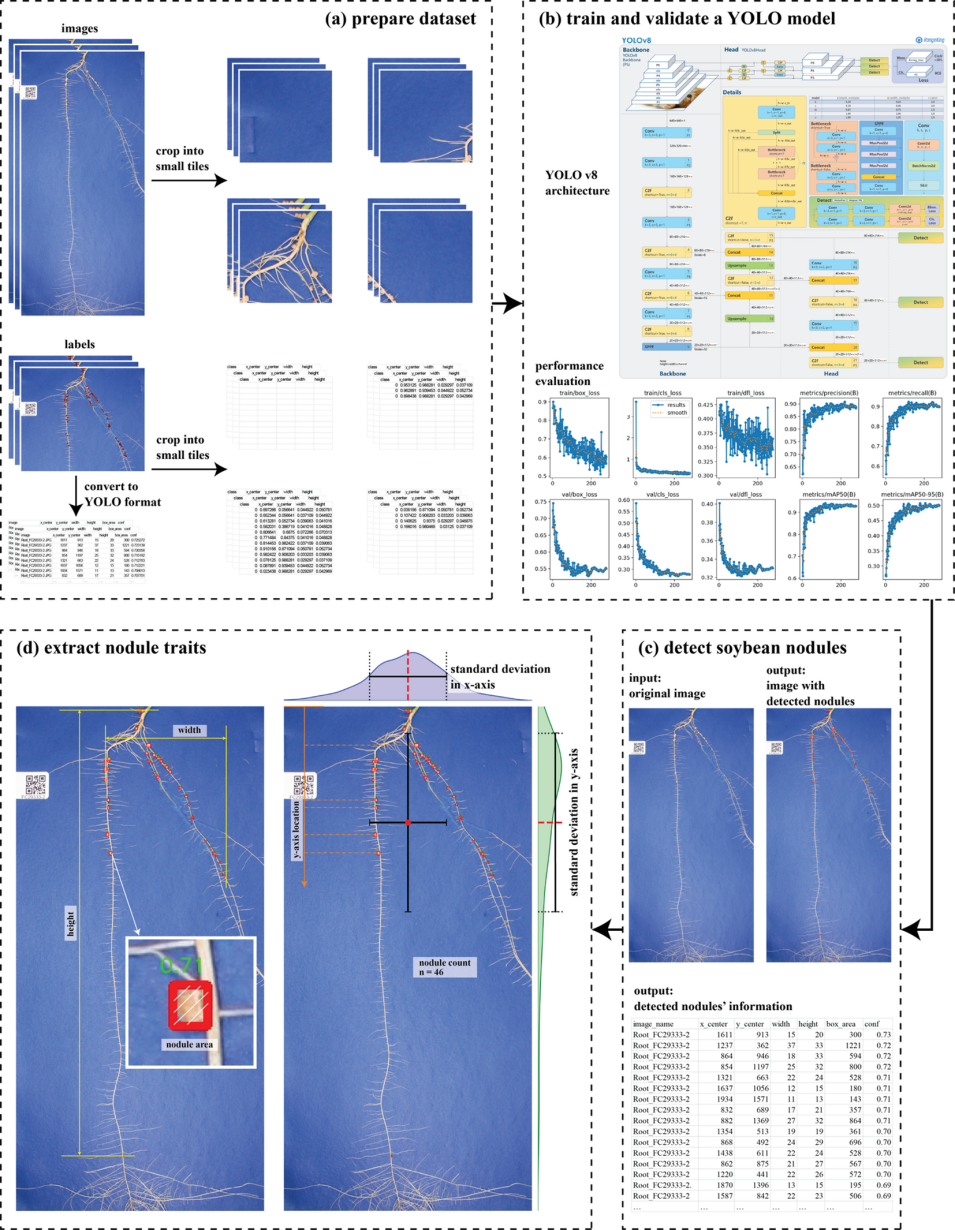
Workflow involved in the development of the pipeline. **(a)** Prepare model training dataset, including cropped images and manual labels with YOLO formatting. **(b)** Train and validate a YOLO-based model. YOLO architecture picture cited from https://github.com/ultralytics/ultralytics/issues/189. **(c)** Detect soybean nodules with trained YOLO-based model and new images. **(d)** Extract nodule traits.

The soybean nodule detection model was developed using a deep learning approach implemented in Python software. This model leverages the capabilities of convolutional neural networks to identify and localize nodules on soybean roots from high-resolution images. Model training and validation were conducted on a high-performance computing platform, run:ai (https://www.run.ai/), using two graphics processing units (NVIDIA A40 GPU) with a memory of 90 GB. The workflow for developing the soybean nodule detection includes the following steps (**Figure 2**). (1) Preparation of the dataset through manual annotation of soybean nodules and cropping of images into smaller patches. (2) Training and validation of the soybean nodule detection model and evaluation of the trained model’s performance using new images. (3) detecting soybean nodules using the trained model. (4) Extraction of nodule-related traits.

To begin with, we manually annotated a total of 70 high-resolution images containing soybean root system with visible nodules. This labelling process was conducted using LabelMe (https://github.com/labelmeai/labelme), a graphical image annotation tool that allow users precise drawing bounding boxes around the object edges. In this case, each individual soybean nodule was labeled with a rectangular box that tightly encompassed the visible edges of the nodule to ensure accurate spatial representation. The annotations were initially saved as JSON file in LabelMe. The files were then programmatically converted to You Only Look Once (YOLO) dataset format, which requires annotations in terms of the normalized coordinates of the bounding box center along with its width and height. The normalization process was performed with respect to the original dimensions of each image, allowing the model to generalize across images of varying resolutions. Those images and manual annotations were used for soybean nodule detection model training and validation.

Next, to reduce the computation memory requirements while maintaining high detection accuracy, the original images (approximately 2,300 pixels by 4,600 pixels) were cropped into small patches with size of 512 pixels by 512 pixels. To minimize the risk of cutting through nodules at the edges of the patches, which could result in partial or missed detections, a padding strategy was conducted. For this, a 64-pixel overlap was applied between adjacent patches along both the horizontal and vertical axes. This overlap ensured that nodules located near patch edges were fully captured in at least one neighboring patch. The width of 64 pixels was selected because the maximum nodule length was 57 pixels, with an average length of 19.93 pixels across all testing images. In parallel with image cropping, the corresponding label files containing the soybean nodule locations were also adjusted. Each bounding box annotation from the original image was translated and mapped to the coordinate space of the relevant image patch. As a result, each small patch had its own set of localized labels, representing only the nodules visible within that patch. This patch-level dataset of images and corresponding annotations was then used to train and validate the soybean nodule detection model more efficiently and accurately.

Then, the soybean nodule detection model was trained and validated based on YOLO v8 architecture, a state-of-the-art deep learning model for object detection developed by Ultralytics (https://docs.ultralytics.com/models/yolov8/). Specifically, we employed the YOLOv8n pretrained model, a lightweight version of the model that balances detection accuracy and computational efficiency. For the dataset split, 70% of original annotated images were randomly selected for model training, while the remaining 30% were reserved for validation. We adopted the YOLO augmentation settings to train a robust model for different lighting and camera angle conditions. The model was trained using cropped image patches with sizes of 512 pixels by 512 pixels. Training was conducted for a maximum of 400 epochs, with a batch size of 4. The model was validated using the default evaluation matrix with losses, precision, recall, mean Average Precision at intersection over union (IoU) threshold 0.5, and mean Average Precision across multiple IoU thresholds from 0.5 to 0.95.

We further evaluated the robustness of the model using 100 new images that were not involved in the training or validation processes. Each image was manually annotated using the labelme tool to create ground truth labels for nodules. Meanwhile, the same set of images was processed by the trained model to generate predicted bounding boxes of nodules. To evaluate the accuracy of the model’s predictions, we computed a confusion matrix for each image by comparing the predicted nodules against the manually annotated ground truth. This matrix includes counts of true positives (correctly predicted nodules), false positives (incorrectly predicted nodules), and false negatives (missed nodules). Using the values from the confusion matrices, we then calculated three key performance metrics: precision, which measures the proportion of predicted nodules that were correct; recall or true positive rate, which reflects the proportion of actual nodules that were successfully detected; and the F1 score, which provides a harmonic mean of precision and recall, offering a balanced view of the model’s performance.

Finally, the location of soybean nodules were predicted for each individual 512 pixels by 512 pixels patch using the best soybean nodule detection model. After obtaining predictions for all patches associated with a given original image, the patches were stitched back together to reconstruct the full image. To refine the final set of predicted nodule locations and remove artifacts caused by patch overlap (since adjacent patches shared a 64-pixel region, a single nodule could be detected multiple times at slightly different positions), two post-processing steps were applied. First, an edge threshold of 5 pixels was used to filter out bounding boxes located too close to the edges of patches. These edge detections were considered unreliable, as the associated nodules may have been partially visible or duplicated across neighboring patches. Any detection falling within 5 pixels of a patch edge was removed to reduce false positives. Second, a Non-Maximum Suppression (NMS) algorithm was applied to remove redundant predictions in overlapping regions between patches. The NMS algorithm compared overlapping bounding boxes and retained only the one with the highest confidence score, effectively eliminating duplicates while preserving the most accurate detection. For the parameters of NMS algorithm, a confidence threshold of 0.2 and an IoU threshold of 0.2 were used to filter duplicated predictions. The relatively low confidence threshold was chosen to retain weak but potentially valid detections. In addition, a low IoU threshold was applied because true nodules are generally spatially separated and rarely overlap in 2D projections. At the conclusion of this process, all refined predictions for each original image were compiled into a comprehensive output file. A CSV file was generated, containing the coordinates of all detected soybean nodules in the context of the full-resolution images. This structured output serves as the basis for downstream analyses, including the extraction of phenotypic traits such as nodule count, spatial distribution, and size, thereby supporting high-throughput phenotyping in soybean nodule research.

Soybean nodule traits were quantitatively extracted based on the predicted bounding boxes generated by the YOLO-based detection model, using computer vision techniques implemented in Python software. The first and most fundamental trait extracted was nodule count, which simply refers to the total number of detected nodules in each image. This metric provides a direct measure of nodule abundance. In addition to counting, several spatial distribution metrics were computed based on the center coordinates of each detected nodule’s bounding box. Specifically, nodule area height was calculated as the vertical distance (along the y-axis) between the topmost and bottommost nodules, measured by the y-coordinates of their center points. This metric reflects the vertical extent or zone within which nodules are distributed on the root. Similarly, nodule area width was calculated as the horizontal span (along the x-axis) between the leftmost and rightmost nodules, based on the x-coordinates of the nodule centers. This measure captures the lateral spread of the nodules. To evaluate how uniformly or irregularly the nodules are distributed, standard deviations of the x- and y-coordinates of all detected nodules were calculated. These provide insights into the spatial dispersion of nodules across the root system. Furthermore, the ratio of the standard deviation in the horizontal axis (x) to that in the vertical axis (y) was computed to assess the relative spread or orientation bias of the nodule distribution. In addition, a total of nine statistical summaries were derived from bounding box area and nodule location in y axis values: minimum, maximum, standard deviation, average (mean), median, as well as the 5th, 25th, 75th, and 95th percentiles. These statistics provide a detailed profile of nodule size variation within each plant sample.

### 2.3 Statistical analysis

Statistical analysis of the dataset comprising 24 nodulation-associated traits, obtained from the newly established phenotyping model, was performed by calculating frequency distributions for each trait, estimating variability using standard deviation, and detecting outliers based on a Z-score threshold of |Z| ≥ 4. Outlier values exceeding this threshold were removed to reduce distortion in trait variance and to ensure data suitability for downstream GWAS. Trait-wise histograms were generated using the rMVP package in R (https://github.com/xiaolei-lab/rMVP) to visualize the distribution and variability of each trait across accessions. Furthermore, to investigate the interrelationships among the phenotypic traits, Pearson correlation analysis was conducted. This analysis was performed using R code adapted from the resource available at https://malouche.github.io/DataVisuWithR/models.html.

### 2.4 Genome-wide association studies

Genotypic data for 187 soybean accessions were obtained using the Illumina Infinium SoySNP50K iSelect BeadChip platform (https://www.soybase.org/tools/snp50k/). The initial dataset contained 41,245 single nucleotide polymorphisms (SNPs). Following quality control procedures, including the removal of SNPs with a minor allele frequency (MAF) below 0.05 to improve data quality and statistical power, a final high-quality genotype dataset comprising 35,149 SNPs was retained for further analysis.

To assess population structure, principal component analysis (PCA) was performed on the filtered SNP dataset using the **rMVP** package in R (https://github.com/xiaolei-lab/rMVP). The number of PCs retained was determined by evaluating the proportion of genetic variance explained by each component. To account for genetic relatedness among accessions, a kinship matrix was generated using the **GAPIT** R package (https://github.com/jiabowang/GAPIT).

GWAS were performed using the rMVP package, which provides memory-efficient computation, enhanced data visualization, and accelerated processing through parallelization (https://github.com/xiaolei-lab/rMVP). The package offers several statistical models, including the general linear model (GLM), mixed linear model (MLM), and the Fixed and Random Model Circulating Probability Unification (FarmCPU), of which the FarmCPU method was selected. This approach was chosen because it offers greater statistical power compared to conventional models such as GLM and MLM and demonstrates computational scalability, with runtime increasing linearly with both the number of individuals and the number of markers. While the association analyses were carried out in rMVP, visualization of the results was conducted using the CMplot R package (https://github.com/YinLiLin/CMplot). Manhattan and quantile–quantile (QQ) plots were generated in CMplot, where Bonferroni correction was applied to adjust p-values and control for false discovery. The conventional Bonferroni-corrected threshold at −log_10_(P) = 5.84 (α = 0.05) is often regarded as overly conservative in soybean due to the extensive linkage disequilibrium present in this species (Hyten et al., 2007). Therefore, in addition to this stringent cutoff, a relaxed threshold of −log_10_(P) ≥ 5 was also applied. This secondary threshold, while less stringent than the Bonferroni correction, is more conservative than those adopted in some previous soybean GWAS reports (Kaler et al., 2017b). Both thresholds were employed in the Manhattan plots to detect significant SNP–trait associations, and the identified loci were subsequently compared with previously reported genomic regions to evaluate overlaps and shared candidate genes. Comparative visualization was performed using MapChart 2.3.2 (Voorrips, 2002). In regions with dense significant SNPs, LD patterns and haplotype blocks were visualized using LDBlockShow, a fast and efficient tool for generating LD heatmaps (Dong et al., 2021).

### 2.5 Identification of candidate genes

To identify candidate genes associated with soybean nodulation, genes located within a 50 kb region upstream and downstream of each significant SNP were extracted using the SoyBase platform (http://www.soybase.org). Further, the Gene Ontology (GO) annotations and enrichment analysis were conducted for the extracted genes to assess their associated molecular functions, biological processes, and cellular components using the GO analysis tool available on SoyBase (https://www.soybase.org/tools/analysis/go.html).

To prioritize candidate genes identified from SNP-associated regions, tissue-specific expression profiles were retrieved from the ePlant database for soybean (https://bar.utoronto.ca/eplant). Expression levels were examined across seven tissues: shoot apical meristem (SAM), flower, green pods, leaves, nodules, roots, and root tips. A heatmap visualizing the expression profiles of the final candidate genes across these tissues was generated using a customized Python script implemented in the Google Colab environment (https://colab.research.google.com/). Expression data for a subset of the candidate genes were not available on the ePlant platform. In such cases, differential expression analysis was carried out using publicly available soybean RNA-Seq datasets (4,085 libraries) accessed through the IPF web server with default parameters (http://ipf.sustech.edu.cn/pub/plantrna/). By integrating these two approaches, database-based expression retrieval and RNA-Seq datasets, we systematically filtered and prioritized candidate genes based on their tissue-specific expression profiles, similar to previous studies (Rathore et al., 2024; Kim et al., 2023).

Further, to evaluate the evolutionary conservation of the identified candidate genes, a comparative orthology analysis was performed using the Venn-Ortho tool. Protein sequences of the soybean candidate genes were compared against the complete proteomes of *Medicago truncatula*, *Phaseolus vulgaris*, *Vigna angularis*, and *Glycine max* using the Venn-Ortho web platform (https://orthovenn2.bioinfotoolkits.net).

In addition to these analyses, a functional protein–protein interaction (PPI) analysis was performed to explore the relationships among the GWAS-identified candidate genes and other biologically relevant interactors. The candidate genes were first queried in the STRING database (https://string-db.org/) to construct an interaction network, which was subsequently visualized and analyzed in Cytoscape. Hub proteins within the STRING-derived network were identified based on maximal clique centrality (MCC) scores using the CytoHubba plugin. In parallel, functional module analysis was conducted using the SoyNet platform (https://www.inetbio.org/soynet/search.php), which predicts gene-level functional associations by integrating candidate genes with significant intermediate nodes. The SoyNet-derived networks were also visualized in Cytoscape, and hub genes within each module were identified based on MCC scores. To further assess the biological significance of the hub genes identified from both approaches, annotation descriptions were extracted from SoyBase (https://soybase.org), and their expression profiles were examined in soybean tissues, including roots and nodules, using the BAR Toronto database (http://bar.utoronto.ca/efp_soybean/).

## 3 Results

### 3.1 Training and validation of the YOLO model for soybean root nodule detection

The soybean nodule detection model was developed and evaluated using a dataset with 70 annotated images. These images were split for training and validation to assess the model’s ability to accurately identify and localize nodules under varying conditions. The model employed the YOLO architecture, which is well-known for object detection capabilities and balance between computing speed and accuracy.

**Figure 3:**
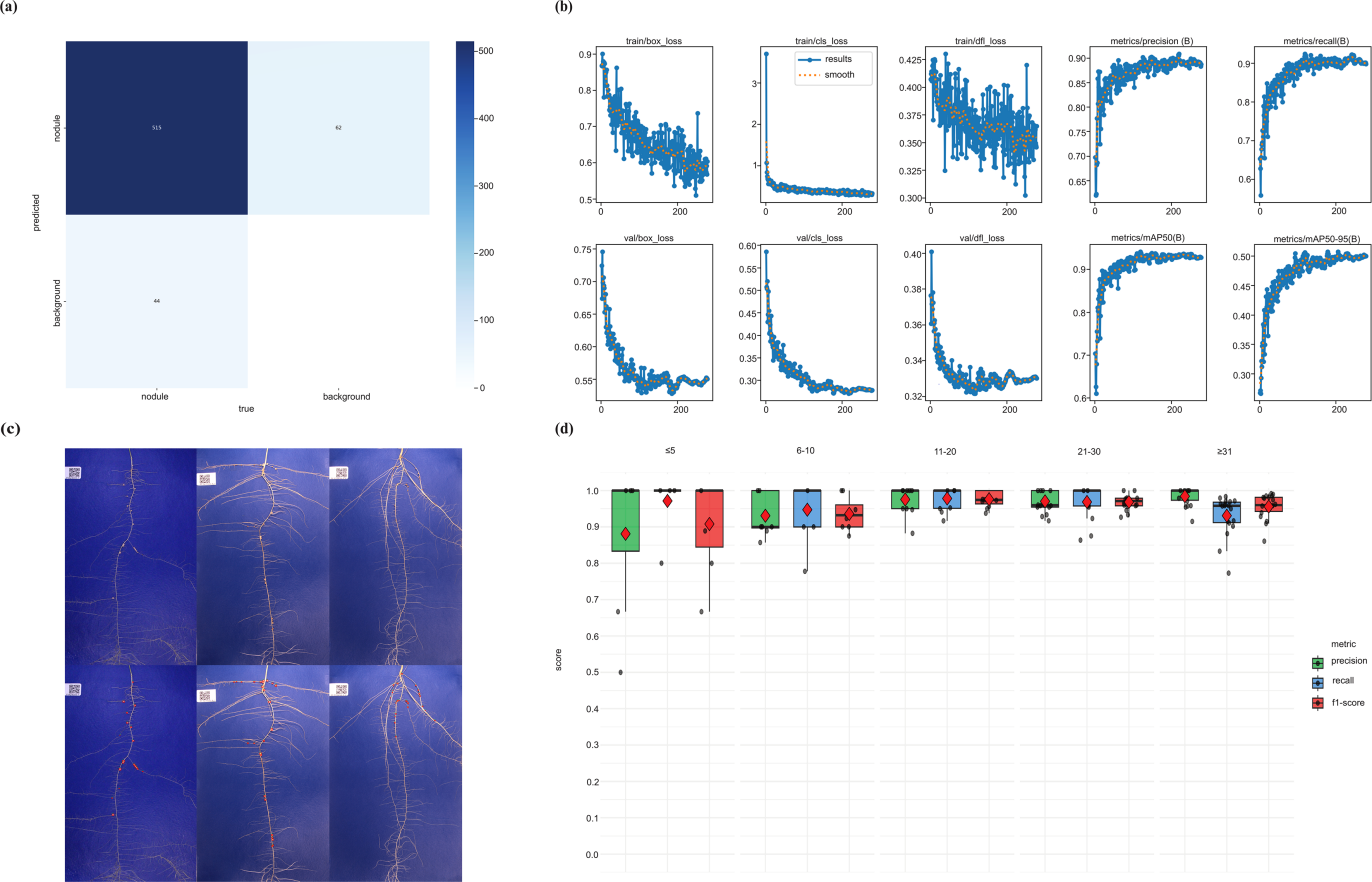
Performance evaluation of the soybean nodule detection model. **(a)** Confusion matrix illustrating classification accuracy across predicted and actual classes. **(b)** Validation performance metrics summarizing the model’s predictive capability. **(c)** Representative output images demonstrating accurate localization of nodules within a complex root background. **(d)** Boxplots showing the distribution of precision, recall, and F1-score across accessions grouped by manual nodule count ranges.

Model validation was conducted through both a confusion matrix and standard object detection performance metrics (**Figures 3a, b**). Based on the validation results, the confusion matrix revealed that the model successfully detected 515 true positives (TP) out of 559 nodules (thus 44 false negatives (FN)), indicating a high number of correctly identified nodules. However, it also produced 62 false positives (FP), where non-nodule regions were incorrectly predicted as nodules, representing missed detections of actual nodules. These results suggest the model has strong detection capability, but with some over-detection and occasional under-detection. To further quantify the model’s performance, precision and recall were calculated. Precision, which measures the proportion of true positive predictions among all predicted positives, indicates how well the model avoids false detections. Recall, on the other hand, reflects the model’s ability to capture all relevant objects. Both metrics showed improvement as training progressed, suggesting effective learning from the dataset. In addition, the model’s accuracy in localizing nodules was assessed using the mean Average Precision (mAP), a standard metric in object detection tasks. The mAP was first computed at an Intersection over Union (IoU) threshold of 0.50 (denoted as mAP@0.50), providing a baseline performance measure. Furthermore, to evaluate the model’s robustness across stricter overlap criteria, the average of the mean Average Precision across multiple IoU thresholds (from 0.50 to 0.95 in steps of 0.05), known as mAP@[0.50:0.95], was also reported. Both mAP@0.50 and mAP@[0.50:0.95] increased steadily as training epochs advanced, reflecting improved bounding box accuracy and overall model generalization. Moreover, the loss curves from training and validation phases consistently decreased with each epoch, further confirming convergence of the model and its ability to minimize error over time. These metrics demonstrate that the YOLO-based detection model is effective and reliable for automatic soybean nodule detection, providing a promising tool for high-throughput phenotyping and trait analysis.

To visualize the detection performance of the YOLO-based model, a representative example image were generated (**Figure 3c**). This output demonstrates the model’s ability to accurately identify and localize nodules on soybean roots, effectively distinguishing them from background features. The image highlights the practical application of the model in detecting varying sizes and densities of nodules across images, serving as a qualitative validation of its real-world usability in root phenotyping pipelines.

In addition, we evaluated the model’s performance using 60 new images that were randomly selected and not included in the training process. For a more refined interpretation, images were grouped into five categories (bins) according to their manually labeled nodule counts: ≤5, 6–10, 11–20, 21–30, and >31 nodules, with the maximum observed nodule count in this category reaching 72. (**Figure 3d**). Within each bin, performance metrics such as precision, recall, and F1-score were computed individually for each image and then averaged to obtain a representative value for that bin. In the ≤5 nodules category, the classifier achieved a precision of 0.8809, recall of 0.9714, and F1-score of 0.9079. Although recall was high, indicating that most true nodules were correctly identified, precision was relatively lower due to the increased influence of false positives in accessions with very few nodules. This reflects a sensitivity of the precision metric in sparse scenarios where even minor misclassifications can disproportionately affect performance. For images containing 6–10 nodules, the model achieved a precision of 0.9308, recall of 0.9472, and f1-score of 0.9358. These values indicate strong performance and demonstrate the model’s ability to reliably detect nodules in low-to-moderate abundance scenarios. In the 11–20 nodule group, the classifier exhibited the highest performance across all bins, with a precision of 0.9754, recall of 0.9782, and F1-score of 0.9761. These values suggest that this range represents an optimal nodule density for the classifier, where it can perform with minimal misclassifications. For the 21–30 nodule bin, the model continued to perform strongly, achieving a precision of 0.9703, recall of 0.9686, and F1-score of 0.9684. In the final bin, which included images with more than 31 nodules, the highest observed nodule count was 72. The classifier attained a precision of 0.9843, which was the highest among all bins, along with a recall of 0.9309 and an f1-score of 0.9559. The slightly reduced recall in this category may be attributed to difficulties in detecting individual nodules within densely clustered regions, where occlusion or overlapping structures can result in under-detection. The average bin-wise performance across all categories was 0.9483 for precision, 0.9592 for recall, and 0.9488 for F1-score. Although these values were marginally lower than the overall (global) metrics, they provide a more detailed characterization of model performance across varying levels of nodule abundance. This analysis demonstrates that the model maintains consistent accuracy and adapts effectively to both sparse and dense nodule distribution conditions.

### 3.2 Automated root phenotyping and analysis of soybean nodulation traits using a YOLO pipeline

**Figure 4:**
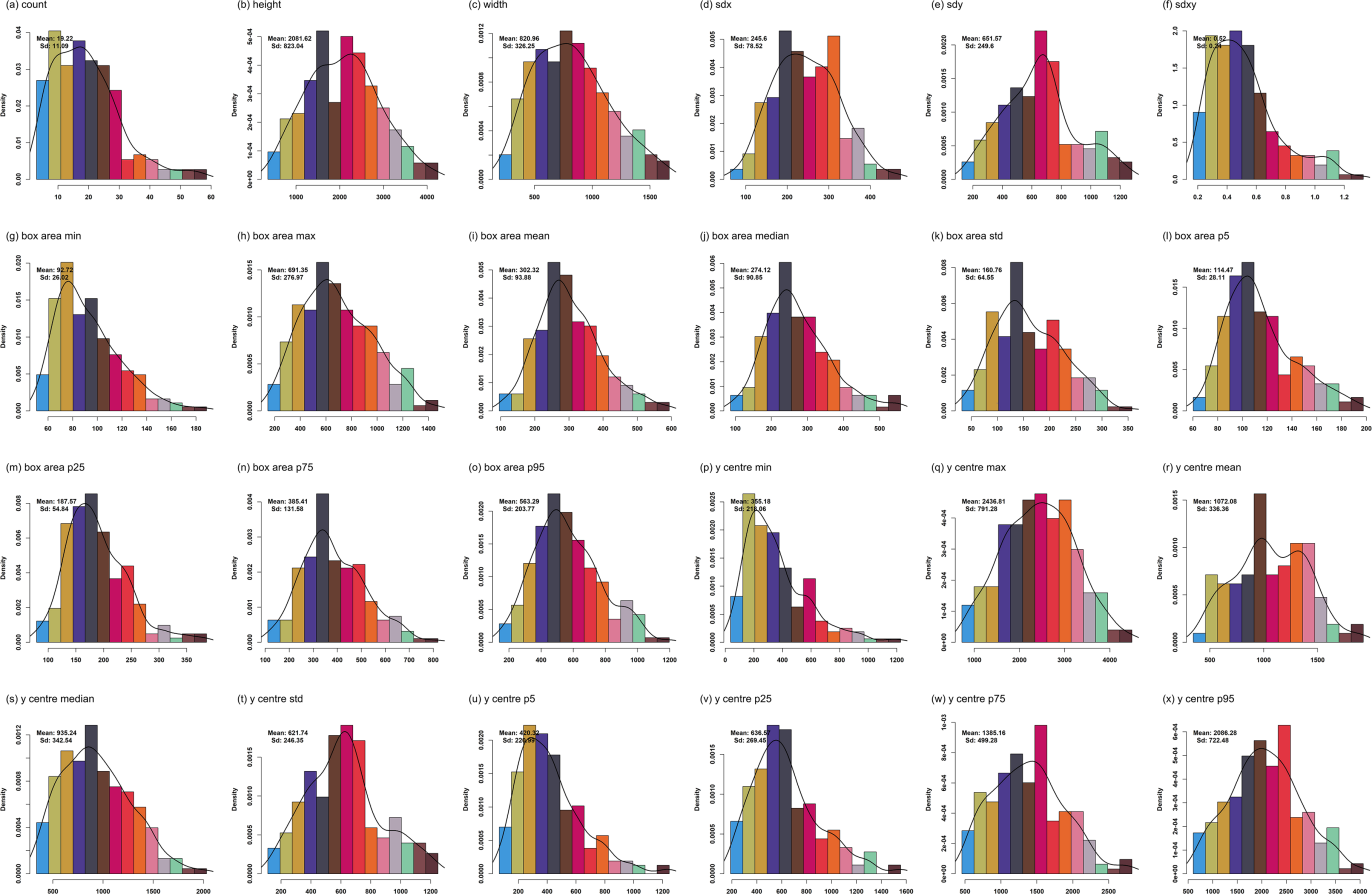
Distribution of soybean nodulation traits. Histograms **(a–x)** show variation in 24 nodulation- and root-related traits across 187 accessions. Trait values are on the x-axis, density on the y-axis, with mean and standard deviation indicated for each trait.

This pipeline was applied to phenotype 187 soybean accessions for subsequent GWAS. To quantify variability in nodulation-related traits among the soybean accessions, the mean, standard deviation, and coefficient of variation (CV%) were calculated for each of the 24 traits, and their frequency distributions were visualized using trait-wise histograms (**Figure 4**). The histograms revealed broad and continuous distributions for most traits, with varying degrees of skewness and dispersion across accessions.

The total number of nodules (count) exhibited a mean value of 19.22 with a CV of 57.69%. Its histogram displayed a right-skewed distribution, characterized by a higher frequency of accessions with low to moderate nodule counts and a smaller proportion of genotypes exhibiting high nodule numbers, reflecting substantial variability in nodulation capacity. The vertical extent of nodulation (height) showed a mean of 2081.62 pixels and a CV of 39.54%, with the histogram indicating a wide distribution spanning approximately 500 to over 4000 pixels. Similarly, nodulation width averaged 820.96 pixels with a CV of 39.74%, and its histogram demonstrated a broad spread with moderate right skewness, indicating heterogeneous lateral expansion of nodulated root systems.

Spatial dispersion of nodules, assessed through the standard deviations of bounding box centers along the horizontal (sdx) and vertical (sdy) axes, showed mean values of 245.60 and 651.57 pixels, with CVs of 31.97% and 38.31%, respectively. The corresponding histograms displayed unimodal but dispersed distributions, highlighting variability in nodule clustering along both axes. The sdxy ratio, which captures directional asymmetry in nodule distribution, had a mean of 0.52 and a CV of 45.64%, with its histogram indicating a wide range of values, reflecting differences in the relative vertical and horizontal dispersion of nodules among accessions.

Variation in nodule size was further characterized using bounding box area metrics. The average nodule area (box area mean) was 302.32 pixels with a CV of 31.05%, and its histogram showed a continuous distribution with a gradual decline toward higher area values. Minimum and maximum box area traits averaged 92.72 and 691.35 pixels, with CVs of 28.06% and 40.06%, respectively. Histograms for these traits revealed broader distributions for maximum box area, indicating greater variability in the upper range of nodule sizes. Percentile-based metrics, including box area p5 (114.47 pixels; CV 24.50%) and box area p95 (563.29 pixels; CV 36.00%), also displayed wide frequency distributions, capturing variability at the lower and upper extremes of nodule size across accessions.

The vertical positioning of nodules, summarized through y-coordinate–based traits, also showed substantial diversity. The y center mean averaged 1072.08 pixels with a CV of 31.37%, and its histogram exhibited a broad distribution spanning shallow to deeper root positions. Among y-center–related traits, y center min displayed the highest CV (61.39%), followed by y center p5 (52.58%) and y center p25 (42.32%). The corresponding histograms for these traits were strongly right-skewed, indicating that while many accessions had nodules concentrated near the upper root regions, a subset showed nodulation extending much deeper along the root axis.

These results reflect broad differences in the vertical distribution patterns of nodules. Overall, traits related to nodule number and spatial positioning, particularly count and y center min, exhibited the highest variability across accessions. These findings underscore the genetic diversity in nodulation phenotypes and highlight key traits for downstream association analysis.

**Figure 5:**
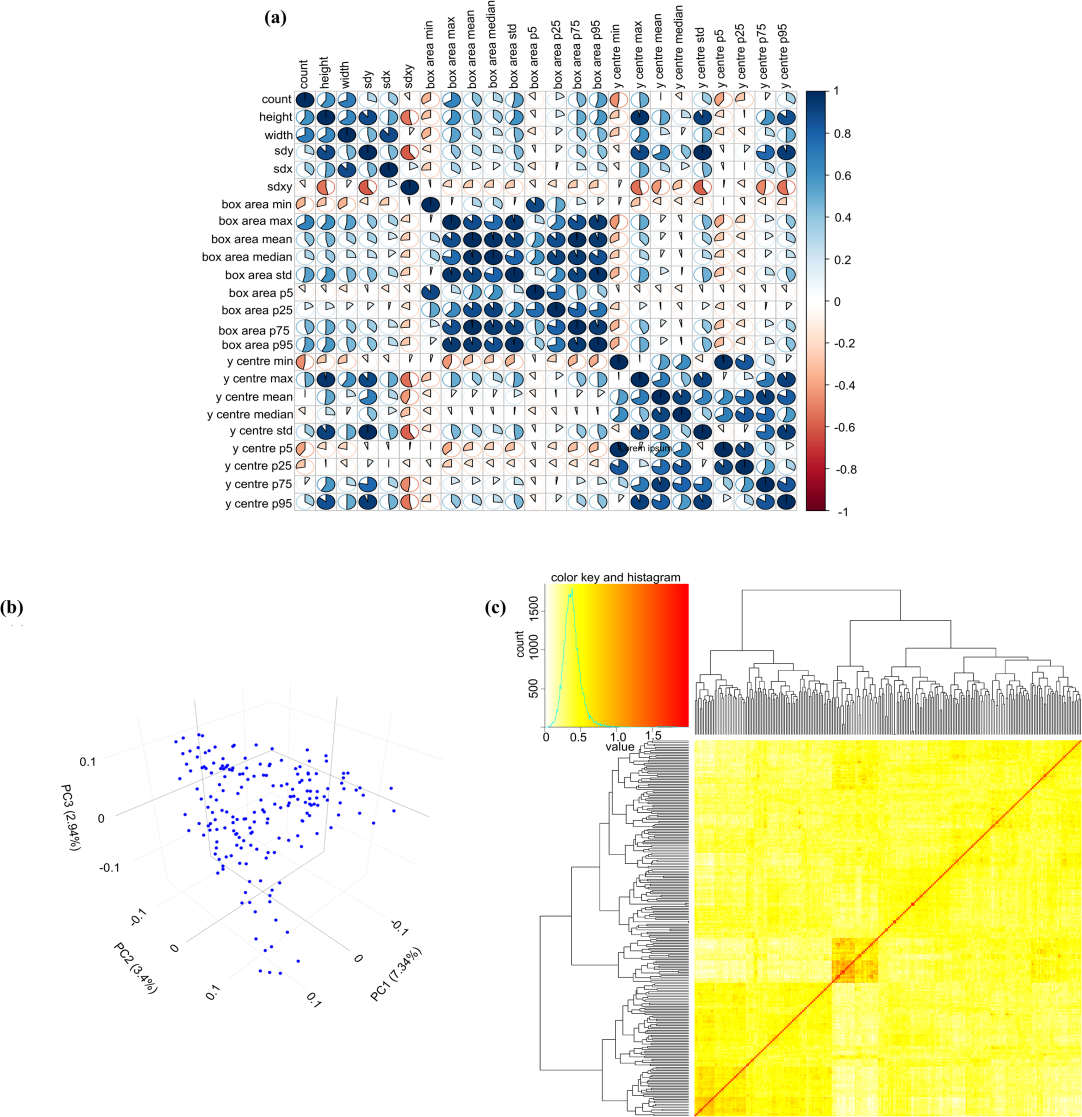
**(a)** Pearson correlation matrix depicting pairwise relationships among 24 nodule traits extracted from 187 soybean accessions. Pie chart segments indicate the direction and magnitude of correlation coefficients (*r*), with blue representing positive associations and red representing negative associations. **(b)** Principal component analysis plot of 187 soybean accessions. **(c)** Kinship matrix heatmap illustrating pairwise genetic relatedness among the soybean accessions.

To explore the interrelationships among nodulation traits, Pearson correlation analysis was conducted across all 24 traits derived from image-based phenotyping. Several strong and interpretable associations were observed (**Figure 5a**). Nodule count exhibited positive correlations with height (r = 0.59) and width (r = 0.69), indicating that plants with greater numbers of nodules tend to develop nodulation zones that are both taller and broader. Count was also strongly correlated with maximum nodule size (box area max; r = 0.66), while its correlations with minimum nodule size (box area min; r = −0.34) and y-center minimum (r = −0.46) were negative. These patterns suggest that accessions with high nodule numbers are characterized by larger nodules and broader nodulation zones, but the lowest vertical positions of nodules and the smallest nodules in the distribution are less represented in such genotypes.

Height was strongly correlated with width (r = 0.64) and vertical dispersion (sdy; r = 0.89.) and showed very strong associations with y-center standard deviation (r = 0.91) and y-center maximum (r = 0.96). This indicates that vertically extended nodulation zones are consistently accompanied by lateral expansion and greater vertical distribution of nodules. Similarly, width correlated strongly with horizontal dispersion (sdx; r = 0.88), highlighting its close relationship with lateral spread of nodules along the root axis.

For size-related traits, box area minimum showed a very strong correlation with box area p5 (r = 0.89), reflecting consistency between the smallest nodules and the lower percentile of the nodule-size distribution, but its relationship with box area maximum was negligible (r = 0.02), indicating independence between the smallest and largest nodules. In contrast, box area maximum was strongly correlated with box area p75 (r = 0.86) and box area p95 (r = 0.94), demonstrating that the upper portion of the size distribution scales consistently with maximum nodule size. These relationships confirm that nodule size traits are internally coherent, with percentile-based and extreme measures capturing complementary aspects of size variation across genotypes.

For y-center traits, y-center maximum was positively correlated with sdy (r = 0.89), showing that greater maximum vertical placement of nodules corresponds to increased vertical dispersion. By contrast, y-center maximum was negatively correlated with box area minimum (r = −0.29), suggesting that genotypes with nodules positioned at higher vertical locations tend to have fewer small nodules. y-center minimum was weakly and negatively correlated with width (r = −0.28), indicating that nodules positioned at the lowest vertical points are less frequently associated with broad lateral expansion.

Overall, the results indicate that soybean plants with extensive nodulation are characterized by broader and taller nodulation zones, greater variability in spatial distribution, and larger nodules (**Supplementary Figure 1**). The observed correlation structure highlights the interdependence of nodulation traits and provides a robust framework for interpreting multivariate trait relationships, thereby supporting downstream analyses such as genome-wide association mapping.

### 3.3 Population structure analysis and GWAS to identify genetic loci associated with nodulation traits

To integrate the obtained phenotypic insights with genomic information, genotype data for the 187 accessions were extracted using the SoySNP50K BeadChip array. PCA was performed to visualize the genetic structure of the panel, revealing clustering patterns consistent with the population structure inferred at K = 3. The first, second, and third principal components (PC1, PC2, and PC3) explained 7.3%, 3.4%, and 2.94% of the total genetic variation, respectively (**Figure 5b**). In addition, a kinship matrix was generated to assess the degree of relatedness among individuals based on allele sharing. A concentrated, red-shaded block in the center of the matrix indicated a subset of individuals with high genetic similarity, reflecting the presence of population stratification within the association panel (**Figure 5c**).

**Figure 6:**
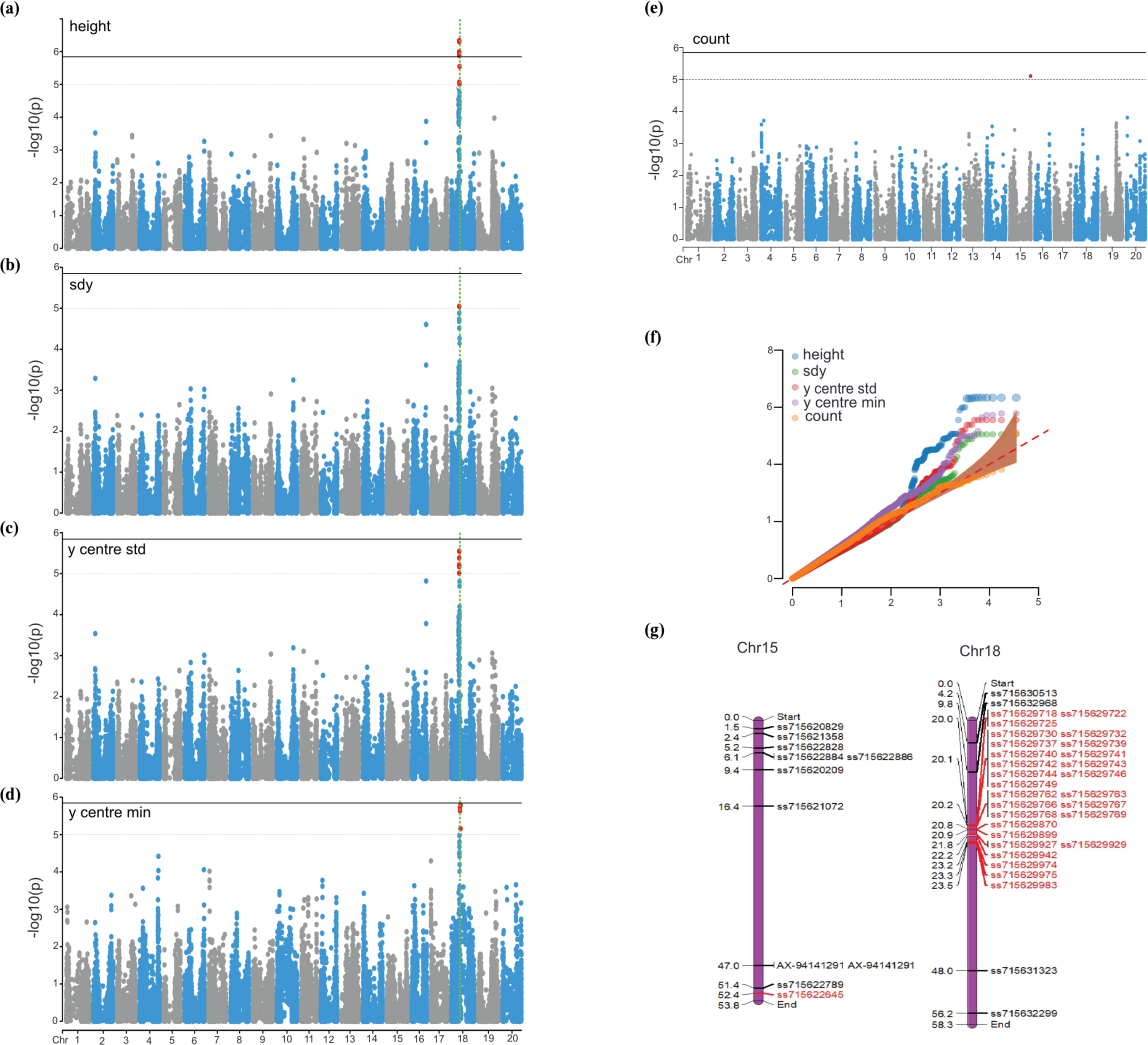
Genome-wide association study (GWAS) results for nodulation-related traits using the FarmCPU model, with −log_10_(*p*) thresholds set at 5.00 and 5.84. **(a)** Manhattan plot for the height trait. **(b)** Manhattan plot for the sdy trait. **(c)** Manhattan plot for y-center standard deviation. **(d)** Manhattan plot for y-center minimum. **(e)** Manhattan plot for nodule count. **(f)** Quantile–quantile (QQ) plots for the corresponding traits, including count, height, sdy, y-center minimum, and y-center standard deviation. **(g)** Comparative chromosomal map showing significant SNPs identified in this study (red) alongside previously reported SNPs (black).

GWAS was performed using the FarmCPU model across all 24 nodulation-related traits extracted from image-based analysis. At the genome-wide significance threshold of −log_10_(p) ≥ 5, a total of 50 significant SNPs were detected, distributed across five traits: height (**Figure 6a**), sdy (**Figure 6b**), y-center standard deviation (**Figure 6c**), y-center minimum (**Figure 6d**), and count (**Figure 6e**). The corresponding quantile–quantile (QQ) plot summarizing all traits is presented in **Figure 6f**. No significant associations were detected for the remaining 19 traits at this cutoff, likely reflecting control by numerous small-effect loci below the detection limit.

In the Manhattan plots (**Figures 6a–e**), two thresholds are displayed: −log_10_(p) = 5, which was used as the primary significance cutoff, and −log_10_(p) = 5.84, corresponding to the Bonferroni correction based on the number of markers tested. While both thresholds are shown for reference, all downstream analyses were conducted using the −log_10_(p) ≥ 5 cutoff, selected to balance statistical rigor with sensitivity and to retain loci of potential biological relevance. A relaxed threshold of −log_10_(p) ≥ 4 was also applied, which identified over 120 SNPs across all 24 traits; however, these loci were treated as supplementary information and excluded from subsequent analyses.

Among the significant loci, the height trait exhibited the largest number of associations (n = 22), all clustered within a genomic interval on chromosome 18 (Chr18) spanning 19.96 Mb to 20.28 Mb. This interval constitutes a QTL region identified in the present study. Fourteen of these SNPs surpassed the stringent threshold of −log_10_(p) ≥ 5.84. Within the same interval, 14 SNPs were associated with y center std and 7 with sdy, and these SNPs were the same as those identified for height. Furthermore, the 7 SNPs associated with sdy were also among the 14 SNPs detected for y center std, reflecting complete overlap across the three traits.

Seven SNPs (ss715629730, ss715629737, ss715629739, ss715629740, ss715629741, ss715629743, and ss715629746) were shared by height, sdy, and y center std, with p-values of 4.63 × 10^-7^, 8.91 × 10^-6^, and 2.82 × 10^-6^, respectively. In addition, five SNPs (ss715629725, ss715629742, ss715629749, ss715629732, and ss715629744) were shared between height and y-center standard deviation. For y-center std, the p-values were 4.15 × 10^-6^ for ss715629725, ss715629742, and ss715629749, and 6.71 × 10^-6^ for ss715629732 and ss715629744, while the corresponding values for height were 4.94 × 10^-7^ and 1.01 × 10^-6^, respectively.

In addition to these overlapping loci, the trait y center min, which reflects the lowest vertical position of nodules on the root, was associated with six SNPs located further downstream on Chr18 (21.7–23.4 Mb), indicating the presence of trait-specific regulatory elements distinct from those influencing vertical spread and dispersion. By contrast, nodule count was linked to a single SNP (ss715622645) on chromosome 15 at 52.4 Mb, pointing to genetic control independent from that of the spatial distribution traits.

**Figure 7:**
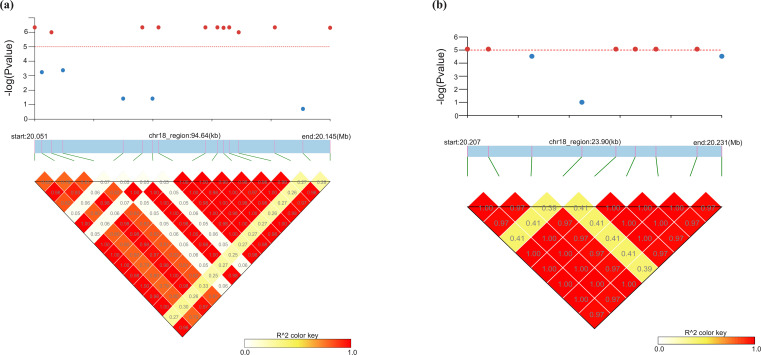
Linkage disequilibrium (LD) patterns among significant SNPs on chromosome 18 detected from the Height trait dataset. **(a)** The first haplotype block covers a 94.64 kb region (20.051–20.145 Mb) and includes SNPs also associated with sdy and y center std. **(b)** The second haplotype block spans 23.90 kb (20.207–20.231 Mb) and contains additional co-associated SNPs. Color intensity represents the LD coefficient (r²) between SNP pairs, with red indicating higher LD values.

SNPs with identical p-values detected for height, sdy, and y center std were further examined through linkage disequilibrium (LD) analysis to assess their correlation. Because all SNPs associated with y center std and sdy were also observed in height, the LD analysis was conducted using the height-associated SNP set. This analysis identified two discrete haplotype blocks within the interval on Chr18. The first block, spanning 20.051 Mb to 20.145 Mb (94.64 kb), contained the majority of SNPs shared among the three traits (**Figure 7a**). The second block, located between 20.207 Mb and 20.231 Mb (23.90 kb), also harbored significant SNPs associated with the same traits (**Figure 7b**). In both blocks, SNPs that exhibited the identical p-values showed perfect LD (r² = 1.00) with respect to other SNPs sharing identical p-values, thereby confirming strong linkage across these loci.

### 3.4 Identification of potential candidate genes associated with nodulation traits and root traits

To identify putative candidate genes associated with nodulation-related traits, we focused on unique SNPs that surpassed the stringent threshold of −log_10_(p) ≥ 5, as detected by the FarmCPU model (**Supplementary Table 1**). For each of these SNPs, genes located within a ±50 kb window were extracted to capture nearby loci potentially involved in trait regulation. Gene annotation and Gene Ontology (GO)-based enrichment information for these genes were retrieved from SoyBase, a comprehensive genomic resource for *Glycine max*. Gene annotation is provided in (**Table 1**) and GO-based enrichment results are in **Supplementary Table 2**. These analyses enabled functional categorization of candidate genes according to the traits with which they were associated. For the trait count, candidate genes were predominantly associated with ATP hydrolysis activity, ubiquitin-dependent protein catabolic processes, carbohydrate metabolic processes, and signal transduction. Several were linked to enzymatic functions in energy metabolism and metal cofactor binding, including Mo-molybdopterin cofactor biosynthesis and the tricarboxylic acid cycle. Key molecular functions included ATP binding, GTP binding, ubiquitin protein ligase activity, citrate synthase activity, GTP 3’,8’-cyclase activity, metal ion binding (iron, zinc), and 4 iron, 4 sulfur cluster binding, as well as stress-responsive roles such as response to cadmium ion.

**Table 1:** List of unique genes located within 50 kb upstream and downstream of significant SNPs across nodulation-related traits.

Gene	Trait	Location	Annotation description
Glyma.15G267900	count	52411888:52412725	Heat-shock protein 20 family member
Glyma.15G268000	count	52425027:52427910	BCS1 AAA-type ATPase
Glyma.15G268100	count	52437445:52440259	Ring zinc finger protein; Ring finger domain
Glyma.15G268200	count	52444126:52445347	Os01g0321850 protein ortholog
Glyma.15G268300	count	52468222:52476393	F16F4.1 protein related; VQ motif containing protein 25
Glyma.15G268400	count	52478227:52484513	Molybdopternin cofactor synthesis protein A
Glyma.15G268500	count	52484411:52494468	Citrate synthase
Glyma.18G136551	height, sdy, y center std	20014181:20014504	N/A
Glyma.18G136900	height, sdy, y center std	20065851:20069824	RNA binding protein; RNA recognition motif
Glyma.18G137000	height, sdy, y center std	20072436:20073978	N/A
Glyma.18G137100	height, sdy, y center std	20075083:20079518	Spermidine/Spermine synthase
Glyma.18G137300	height, sdy, y center std	20135273:20139045	Aldehyde dehydrogenase-related
Glyma.18G137500	height, sdy, y center std	20155371:20159109	Xyloglucan endo-transglycosylase (XET) C-terminus
Glyma.18G137402	height, y center std	20155626:20155880	N/A
Glyma.18G136400	height, y center std	19948616:19950687	General transcription factor 2-related zinc finger protein
Glyma.18G136500	height, y center std	19954838:19960788	Osmotic stress potassium transporter
Glyma.18G139700	height	20933193:20937663	Enoyl-COA hydratase related
Glyma.18G137600	height	20198537:20202665	Os01g0321850 protein ortholog
Glyma.18G137700	height	20233930:20245809	TOLL like receptor
Glyma.18G138000	height	20266115:20268505	Polygalacturonase 1 beta like protein 2
Glyma.18G138900	height	20732711:20737334	Proteasome subunit alpha/beta
Glyma.18G139000	height	20772653:20776228	Putative (DUF220) related
Glyma.18G139100	height	20776243:20784499	Pantothenate kinase
Glyma.18G143800	y center min	23186017:23186565	MADS box protein
Glyma.18G144700	y center min	23466527:23467924	AP2 domain class transcription factor
Glyma.18G141200	y center min	21853386:21875099	Homeobox protein transcription factors
Glyma.18G141400	y center min	21862154:21865585	N/A
Glyma.18G141500	y center min	21873692:21878379	Receptor-like serine/Threonine protein kinase SD1-8
Glyma.18G142200	y center min	22211440:22213899	Replication factor A 1, RFA1
Glyma.18G143900	y center min	23293200:23300870	Arginine/serine rich protein PNISR

The genomic region shared by height, sdy, and y center std contained genes functionally linked to alternative mRNA splicing via the spliceosome, polyamine biosynthesis (including spermidine biosynthetic pathways), response to cadmium stress, aldehyde dehydrogenase activity, and cell wall modification through xyloglucan metabolism. Several genes were associated with potassium ion transport and potassium ion transmembrane transporter activity, suggesting potential roles in ion homeostasis during nodulation. Molecular functions included RNA binding, protein binding, zinc ion binding, ATP binding, hydrolase activity (O-glycosyl bond hydrolysis), and xyloglucan:xyloglucosyl transferase activity, reflecting diverse catalytic, structural, and regulatory capacities.

In addition to these shared loci, height-specific candidate genes were enriched in menaquinone biosynthesis, protein phosphorylation, protein desumoylation, proteolysis, and jasmonic acid biosynthesis. Molecular functions included protein serine/threonine kinase activity, transmembrane receptor protein kinase activity, SUMO-specific endopeptidase activity, endopeptidase activity (including threonine-type), monoatomic cation channel activity, pantothenate kinase activity, and additional catalytic activities involving ATP binding, hydrolase activity, and metal ion binding.

For y center min, candidate genes were primarily associated with regulation of DNA-templated transcription, meristem maintenance, and hormone-mediated pathways, including auxin response, gibberellic acid signaling, and ethylene-activated signaling. Additional processes included embryonic pattern specification, cotyledon development, cell cycle regulation, and response to ozone. Several genes participated in reactive oxygen species metabolism and protein autophosphorylation, indicating roles in stress signaling and developmental regulation. At the molecular level, functions encompassed DNA-binding transcription factor activity (RNA polymerase II-specific), transcription factor binding, sequence-specific DNA binding, protein dimerization activity, kinase activity (including protein serine/threonine kinase activity), ATP binding, and metal ion binding. A subset of genes was further implicated in telomere maintenance, DNA replication, homologous recombination, and nucleotide-excision repair.

In this study, 45 genes were shortlisted based on GWAS results, of which 30 were identified as unique. To investigate their expression profiles, publicly available transcriptomic resources were utilized. The BAR Toronto ePlant soybean database was initially accessed; however, expression data were unavailable for 13 genes. To address this gap, RNA-Seq data from 4,085 soybean transcriptome libraries were retrieved using the IPF web server. Despite the broader coverage of this dataset, expression information for two genes remained inaccessible.

**Figure 8:**
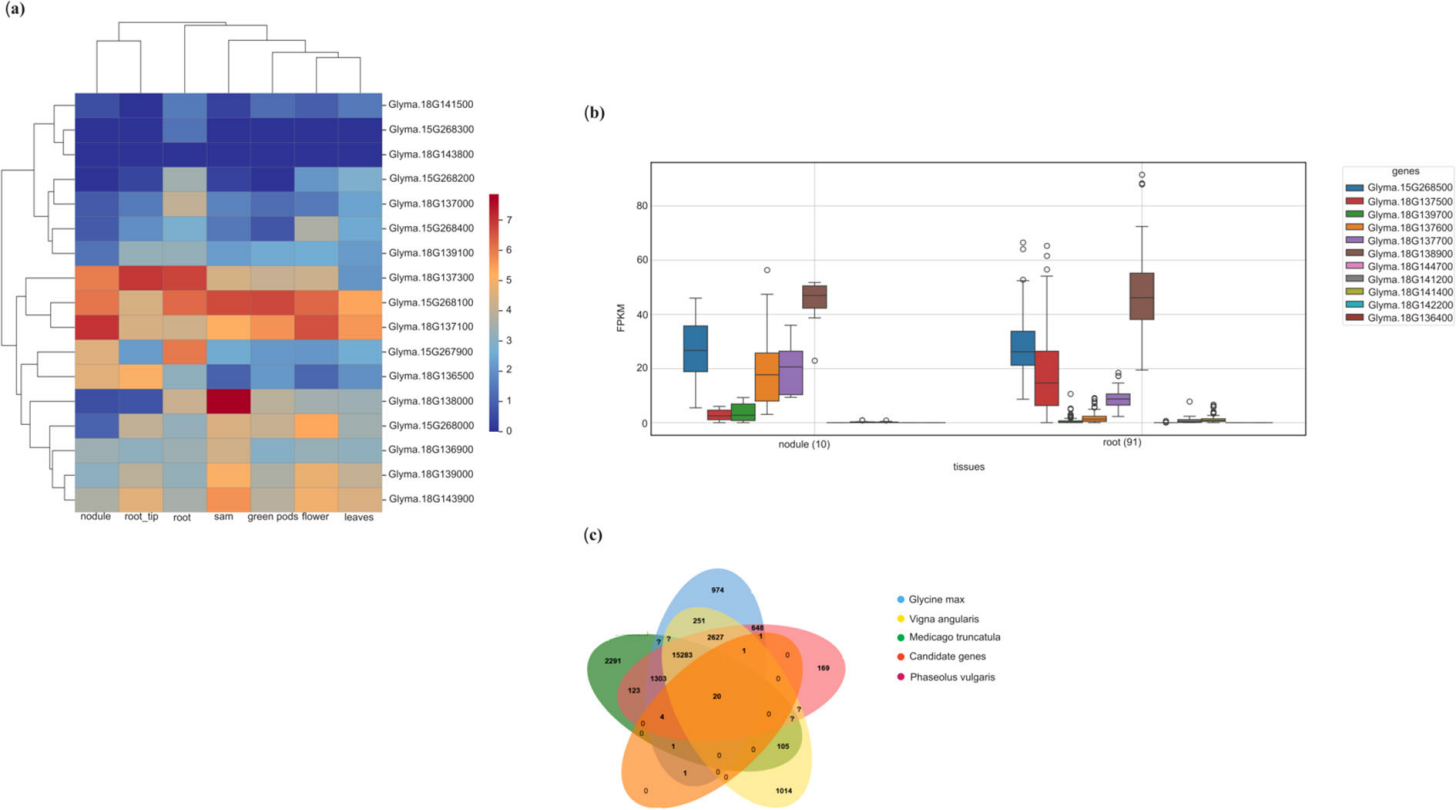
**(a)** Tissue-specific expression heatmap of 17 candidate genes identified through GWAS. Expression data were retrieved from the BAR Toronto ePlant soybean database and visualized on a heatmap. The color gradient represents log-transformed expression levels (0–8) across diverse tissues, including root tips, roots, nodules, leaves, green pods, flowers, and shoot apical meristem (SAM). **(b)** Differential expression analysis of additional candidate genes in root and nodule tissues, performed using RNA-Seq data from over 4,000 soybean transcriptome libraries. These genes correspond to those for which expression data were not available in the BAR Toronto database. Expression values are shown as FPKM in box plot format. **(c)** Comparative orthologous cluster analysis of 28 candidate genes using OrthoVenn2. The analysis included four legume species, *Glycine max*, *Medicago truncatula*, *Phaseolus vulgaris*, and *Vigna angularis*. The Venn diagram illustrates shared and unique orthologous gene clusters among the species, showing 20 clusters conserved across all four and additional clusters unique to individual or subsets of species.

Among the 17 candidate genes with expression data available in the BAR Toronto database, expression patterns were visualized using a heat map (**Figure 8a**). Five genes exhibited relatively higher expression in both root and nodule tissues: *Glyma.15G267900*, a heat shock protein 20 family member; *Glyma.15G268100*, a RING zinc finger protein containing a ring finger domain; *Glyma.18G137300*, an aldehyde dehydrogenase-related protein; *Glyma.18G137100*, a spermidine/spermine synthase; and *Glyma.18G136500*, an osmotic stress potassium transporter.

Analysis of the RNA-Seq compendium comprising more than 4,000 soybean libraries identified two additional genes with notably high expression in root tissue (**Figure 8b**). These included *Glyma.15G268500*, a citrate synthase, and *Glyma.18G138900*, a proteasome subunit alpha/beta.

To evaluate the genomic integrity of the shortlisted candidate genes, a comparative genomic analysis was performed using four closely related species: soybean (*Glycine max*), barrel medic (*Medicago truncatula*), adzuki bean (*Vigna angularis*), and common bean (*Phaseolus vulgaris*). Across these species, 15,283 core gene clusters were identified, of which 974 were unique to soybean. For the candidate gene comparison, 28 root tissue-specific genes were considered; however, three genes were excluded due to the absence of corresponding protein sequence information in UniProt. Analysis of these genes against the four selected species revealed no unique gene clusters. When the comparison was expanded to barrel medic (*Medicago truncatula*), adzuki bean (*Vigna angularis*), and common bean (*Phaseolus vulgaris*), 20 core gene clusters were identified, representing 71.4% (20/28) of the candidate genes. This finding suggests that of these genes have conserved orthologs across related legume species (**Figure 8c**). Functional classification of the 20 core genes indicated their involvement in a wide range of biological processes.

**Figure 9:**
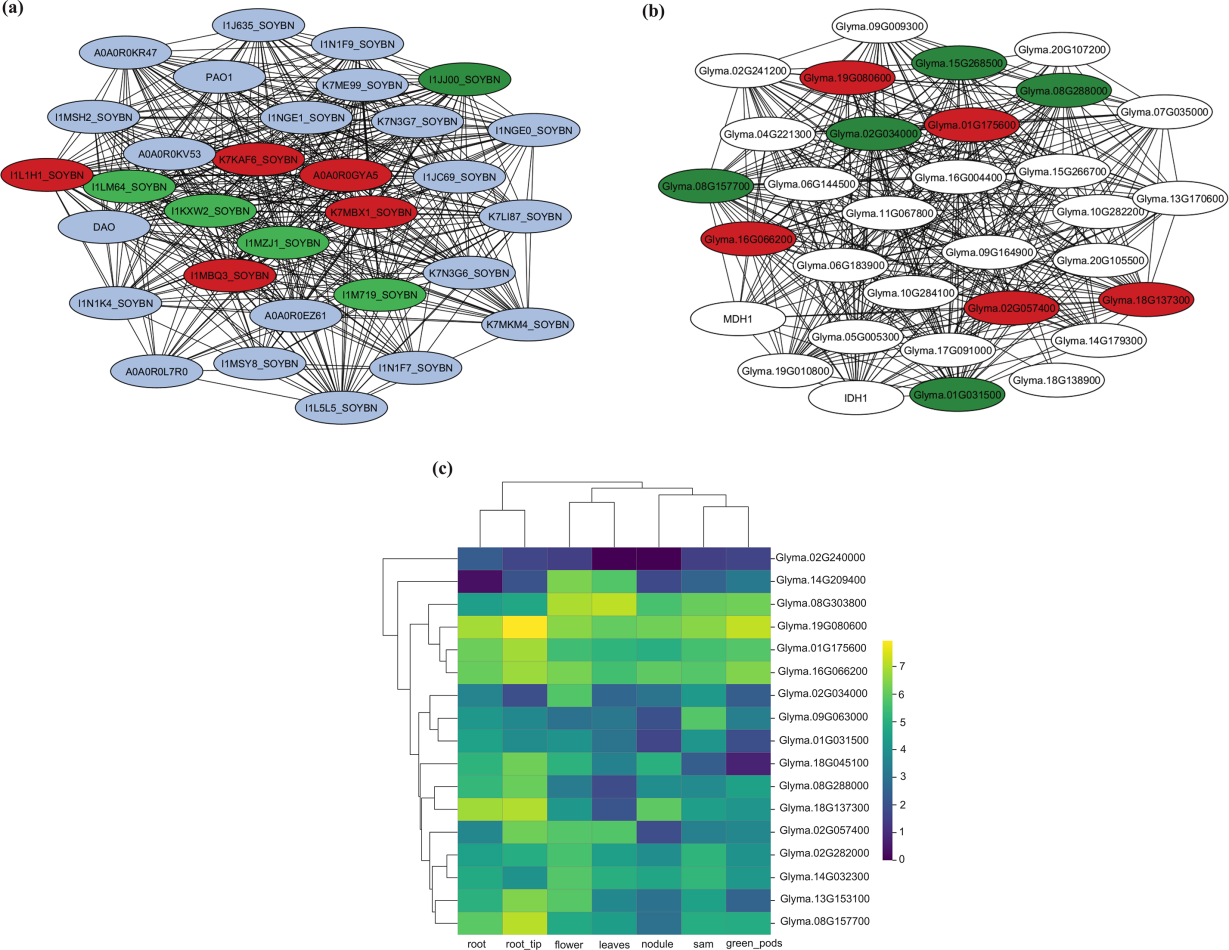
**(a)** Protein–protein interaction (PPI) network of GWAS-derived candidate genes constructed using the STRING database. Hub genes were identified based on MCC scores with the CytoHubba plugin in Cytoscape. **(b)** Functional module network of GWAS-derived candidate genes generated using the SoyNet ‘Find Functional Modules’ tool. Network topology was visualized in Cytoscape, and hub genes were identified based on MCC scores. **(c)** Heatmap showing tissue-specific expression profiles of hub genes identified from both protein–protein interaction and functional module network analyses. Gene expression levels were visualized across multiple soybean tissues, including roots, nodules, leaves, flowers, pods, and shoot apical meristem.

To explore the regulatory potential of the GWAS-derived candidates and place them within a broader biological context, an integrated PPI and network topology analysis was performed. A PPI network was constructed using the STRING database, and hub genes were identified based on MCC scores calculated with the CytoHubba plugin in Cytoscape. This analysis identified ten hub proteins: I1KXW2_SOYBN, I1JJ00_SOYBN, I1M719_SOYBN, I1LM64_SOYBN, I1MZJ1_SOYBN, K7KAF6_SOYBN, I1MBQ3_SOYBN, I1L1H1_SOYBN, K7MBX1_SOYBN, and A0A0R0GYA5, all of which were annotated as flavin monoamine oxidase genes (**Figure 9a**). Among the GWAS-derived candidates, two genes (I1N1F7_SOYBN and I1N1F9_SOYBN) were also present in the PPI network. Although these did not rank within the top 10 hubs, both were embedded in densely connected modules enriched for metabolic and signaling functions. The remaining nodes represented predicted functional interactors, thereby providing a broader biological context for the GWAS candidates.

In parallel, functional module prediction was conducted using the SoyNet “Find Functional Modules” tool with the set of GWAS-derived candidate genes. The resulting network contained 38 nodes organized into several densely connected modules. Only two GWAS-mapped candidates, *Glyma.15G268500* and *Glyma.18G137300*, were present in the SoyNet database. Notably, *Glyma.18G137300* ranked among the top 10 nodes based on degree centrality, indicating a high level of connectivity within the network. Additional hub genes identified in this analysis included *Glyma.08G157700*, annotated as citrate synthase; *Glyma.02G034000, Glyma.08G288000*, and *Glyma.01G031500*, annotated as aldehyde dehydrogenase-related proteins; *Glyma.02G057400* and *Glyma.01G175600*, annotated as succinate dehydrogenase flavoprotein subunits; and *Glyma.16G066200* and *Glyma.19G080600*, annotated as succinate dehydrogenase iron-sulfur proteins. Although these genes were not detected directly through GWAS, they represent strong functional interactors of the GWAS candidates and form tightly linked modules that may participate in nodulation-related pathways (**Figure 9b**). Together, the PPI and SoyNet analyses identified both direct GWAS-mapped candidates and functionally connected hub genes, offering complementary insights into the regulatory landscape underlying nodulation traits.

To further evaluate their expression, profiling of the hub genes was conducted using the BAR Toronto ePlant database, with a focus on transcript abundance in root and nodule tissues. Among the 20 hub genes (10 from the PPI network and 10 from the SoyNet module network), expression data were unavailable for two genes, and two overlapped with those already included in the main candidate set. The remaining 16 hub genes were examined for tissue-specific expression, of which four showed consistently elevated transcript accumulation in root and nodule tissues (**Figure 9c**). Specifically, *Glyma.19G080600, Glyma.01G175600, Glyma.16G066200*, and *Glyma.08G303800* displayed high expression in both root and nodule tissues.

## 4 Discussion

### 4.1 Pipeline efficiency, limitations and future applications of the YOLO-based phenotyping pipeline

YOLO is a high-performance object detection framework widely recognized for its end-to-end detection capability and accuracy (Redmon et al., 2016). Unlike traditional region-based approaches that involve multiple processing stages, YOLO employs a single convolutional neural network to simultaneously predict bounding boxes and class probabilities, enabling rapid and efficient detection across diverse object scales and complexities.

The development of a YOLO-based soybean nodule phenotyping pipeline involves several preparatory steps before model training, including seed germination for seven days, root inoculation with *Bradyrhizobium japonicum*, hydroponic cultivation for twenty-one days, and imaging of individual roots. Because the plants were grown under controlled hydroponic conditions, root cleaning prior to imaging was unnecessary, in contrast to field-grown plants (Jubery et al., 2021).

Once the model was trained, the YOLO based pipeline can be deployed for inference with substantially lower computational requirements, even without GPU. In addition, the pipeline substantially reduced the time and labor required for nodule phenotyping compared to manual annotation. On a single GPU with 45 GB memory, the system processed a batch of 50 root images in 37 seconds, whereas manual annotation of the same set required approximately 2 hours and 20 minutes. This represents an efficiency gain of more than two hundred fold. In comparison, earlier image analysis pipelines reported processing times of 2 to 3 minutes per image in one study and approximately 20 minutes per image in another (Jubery et al., 2021; Remmler et al., 2014). Beyond throughput, the system also demonstrated strong predictive performance, achieving a precision of 0.94, a recall of 0.95, and an F1 score of 0.94 for nodule detection, supporting its reliability for large-scale applications. Model performance was further evaluated across multiple nodule density bins, where it consistently maintained accuracy across varying levels of nodulation. Notably, in the highest density category, where nodule counts reached up to 72 per plant, the system continued to perform robustly, demonstrating its capability to handle relatively dense nodulation conditions (**Supplementary Figure 2**). It is important to note that the current evaluation bins reflect the distribution of the test dataset rather than the full biological range of nodulation. The relatively reduced accuracy observed at higher nodule counts can be attributed to increased object density and occlusion, which inherently complicate detection. This limitation can be addressed in future work by incorporating additional high-density training samples and further optimizing detection parameters to improve model performance under highly crowded conditions.

In addition to efficiency and accuracy, the pipeline expands the scope of trait characterization. A total of 24 traits were quantified, encompassing not only nodule number and area but also spatial distribution along vertical and horizontal axes, variation in nodule size, and positional distribution along the y axis. Such multidimensional trait extraction is valuable because nitrogen fixation efficiency in soybean and other legumes depends on more than nodule number alone, with spatial arrangement and size distribution also playing key roles (Remmler et al., 2014). Unlike previous three dimensional imaging systems that primarily estimated nodule number and area (Jubery et al., 2021), this two dimensional approach provides a broader and more comprehensive dataset for downstream analyses.

The robustness of the pipeline was further demonstrated under controlled hydroponic conditions. Because plants were cultivated in pathogen free environments, early nodule like structures induced by pests such as soybean cyst nematodes (*Heterodera glycines*) were absent, thereby eliminating a potential source of misclassification. The system was also evaluated for potential errors arising from QR coded accession labels, and no false classification of printed dots as nodules was observed. Together, these features underscore the robustness of the developed system and its suitability for generating reliable phenotypic data to support genomic analyses.

Building on these strengths, the pipeline can be extended beyond stationary imaging setups through integration with portable imaging devices, thereby enabling rapid phenotyping in controlled environments (Martinez-Alpiste et al., 2022). With its high accuracy, processing speed, and comprehensive trait coverage, the system represents an effective and scalable solution for large scale agricultural research and breeding programs. Although recent advances have improved methods for evaluating root architecture and nodulation traits (Falk et al., 2020**;** Bucksch et al., 2014), incorporating these traits into high throughput assessments has remained a major challenge. The present pipeline addresses this limitation by enhancing the ability to identify and characterize genetic factors underlying a wide range of nodule related traits. Finally, this framework has practical implications for crop improvement. By enabling the assessment of genotypes with contrasting nodulation performance under diverse biotic and abiotic conditions, while accounting for genotype by environment interactions, the pipeline provides a valuable tool for selecting superior accessions. The markers, candidate genes, and accessions identified through this approach can be leveraged in breeding programs aimed at improving symbiotic nitrogen fixation and crop performance (Peixoto et al., 2017; Falk et al., 2020). In addition to these applications, the pipeline could also be adapted for three dimensional root imaging datasets, as nodulation patterns in soybean are consistent irrespective of whether roots are captured in two or three dimensions, thereby extending its utility for advanced phenotyping studies. However, the current model was developed under controlled experimental conditions and has not yet been validated on dense, highly occluded root systems typically observed in field-grown plants. Consequently, its performance under such complex conditions remains to be systematically evaluated in future studies. Additionally, in this study, root nodule area was estimated from two-dimensional images, which inherently lack depth information. As a result, overlapping nodules and complex root architectures may lead to occlusion, potentially causing underestimation of true nodule size. While our imaging setup was designed to minimize these effects, some degree of inaccuracy is unavoidable without multi-angle or three-dimensional (3D) imaging approaches. Although 3D methods could enhance measurement accuracy, they typically involve greater complexity and reduced throughput. Therefore, our 2D-based approach provides a practical balance between accuracy and scalability, enabling consistent and reproducible measurements across large sample sets.

For deployment in other legume crops, the pipeline should first be validated using two dimensional images to ensure reliable performance before large-scale application. Nematode infections, such as those caused by root knot nematodes (*Meloidogyne* spp.) or soybean cyst nematode, can produce gall-like structures that closely resemble nodules and may lead to misclassification. In this study, all plants were grown under hydroponic conditions and were free of pathogenic infections, so nematode induced structures were not present in the training dataset. As a result, the model has not learned to tell true nodules from pathogen-induced galls, and using infected plants could cause incorrect identification. To avoid this risk, future applications should use infection free plants. In addition, because all plants were imaged on blue blotter paper and the same background was used during model training, keeping similar imaging conditions is recommended. Using blue blotter paper or a similar blue background will help maintain uniformity and improve detection accuracy.

### 4.2 Biological significance of GWAS-identified loci underlying soybean nodulation

In this study, in addition to pipeline development, its utility was further demonstrated through downstream application by integrating the phenotyping outputs with GWAS using a panel of 187 soybean accessions. While earlier GWAS on nodulation have generally been limited to fewer than seven traits, often quantified manually (Fan et al., 2025), the present study employs a fully automated image-based phenotyping approach to capture a broader suite of traits. This integration of deep learning–based phenotyping with GWAS represents a significant advancement in high-throughput, image-enabled plant trait analysis, providing a reliable, rapid, and scalable method for nodule quantification and genetic mapping in soybean and potentially other legumes.

In the study, significant phenotypic variation was observed among soybean landraces for 24 nodulation-associated traits. The highest CV was observed for traits related to nodule spatial distribution, including y center min, y center p5, count, and sdxy, indicating wide phenotypic diversity in nodule positioning and spatial traits among the soybean genotypes. GWAS analysis was conducted using the FarmCPU model, where the choice of significance threshold is a critical factor in detecting reliable and meaningful associations across the genome. In soybean, the application of a strict Bonferroni correction with an alpha value of 0.05 is often considered overly conservative when linkage disequilibrium among genetic markers is high, as is typically the case for this species (Hyten et al., 2007). Consequently, numerous GWAS studies in soybean have adopted less stringent thresholds, below the strict Bonferroni-adjusted value, to facilitate the detection of meaningful associations. For example, GWAS for seed yield and related traits used a threshold of 4.5 (Priyanatha et al., 2022), canopy wilting employed a threshold of 4 (Steketee et al., 2020), seed flooding tolerance used 3.5 (Sharmin et al., 2021), and seed size applied a threshold of 5 (Wang L et al., 2024). Similarly, GWAS for seed number per pod employed both a threshold of 5 and the strict Bonferroni-adjusted value of 5.93 (Wang Q et al., 2024), while agronomic traits were analyzed with a threshold of 5 (Yang C et al., 2021), and seven root traits were studied using thresholds of 4 and 5 (Kim et al., 2023). In line with this approach, the present study applied a −log_10_(p) threshold of 5 for GWAS analysis, which is slightly less stringent than the strict Bonferroni-adjusted value of 5.84.

Using a minimum threshold of −log_10_(p) ≥ 5, significant SNP associations were detected for only 5 of the 24 evaluated traits. The strongest genomic signal was observed on chromosome 18, where a dense cluster of 22 SNPs associated with height coincided with 7 SNPs for sdy and 14 SNPs for y-center std, all of which were identical to the loci mapped for height. The extensive overlap of SNPs across these three traits aligned closely with their strong phenotypic correlations (r = 0.89. for height–sdy, r = 0.91 for height–y-center std, and r = 0.99 for sdy–y-center std), suggesting that a shared genetic basis regulates the vertical and horizontal extent of nodulation. Haploblock analysis further revealed complete linkage disequilibrium (r² = 1) among the SNPs in this region, supporting the presence of a QTL with pleiotropic effects on multiple nodulation traits. The convergence of statistical association, phenotypic correlation, and LD evidence highlights this interval as a key regulatory hotspot controlling nodulation patterning in soybean.

Because significant SNPs were detected on both chromosomes 15 and 18, comparisons were made with previously reported loci to assess overlap with earlier findings (Araya et al., 2023; Huo et al., 2019; Fan et al., 2025). On chromosome 15, SNP ss715622789 at position 51,424,187 bp had previously been associated with nodule count and was located approximately 1 Mb from the SNP identified for the same trait in the present study. On chromosome 18, although several SNPs have been reported for nodulation-related traits in prior studies, these were located far from the genomic interval identified here, underscoring the novelty of the QTL detected for novel traits in this analysis (**Figure 6g**).

### 4.3 Functional and comparative insights into GWAS-derived candidate genes

In the present study, several candidate genes located near significant SNPs were identified that provide important insights into the genetic regulation of soybean nodulation. One such gene, *Glyma.15G268100*, positioned near SNPs associated with nodule count, encodes an E3 ligase with a zinc finger domain and showed high expression in root and nodule tissues. E3 ubiquitin ligases are well known to play critical roles in nodulation, particularly RING-finger proteins that regulate either nodule initiation or the infection process (Hervé et al., 2011). For example, the *LjnsRING* gene in *Lotus japonicus* encodes an E3 ligase with a RING-finger domain and has been associated with the nodulation process (Shimomura et al., 2006). Similarly, *LjPUB13*, another E3 ligase in *L. japonicus*, interacts with Nod factor and positively regulates the early stages of nodulation (Tsikou et al., 2018). Broader studies have also outlined the role of E3 ligases in nodulation across legumes (Li H et al., 2024), while in soybean, E3 ligase genes have been proposed as candidates for symbiotic compatibility with rhizobia (Teraishi et al., 2025). Taken together, the functional annotation and tissue-specific expression of *Glyma.15G268100* strongly support its candidacy for regulating rhizobial infection and nodule development.

Another promising candidate, *Glyma.15G267900*, located near significant SNPs for the count trait, encodes the SF5 small heat-shock protein of the HSP20 family. This gene also exhibited high transcript accumulation in root and nodule tissues. Functional parallels can be drawn with *GmHSP17.1*, whose overexpression during nodule development increases both nodule number and size, while knockdown reduces nodulation capacity (Yang Z et al., 2022). The similarity in expression and annotation suggests that *Glyma.15G267900* may play a comparable role in supporting nodule initiation and development in soybean.

Further, two additional genes, *Glyma.18G137100*, located near SNPs for height, sdy, and y-center standard deviation, and *Glyma.08G303800*, identified through interaction analysis were both annotated with functions in the polyamine biosynthetic pathway. Both genes demonstrated high expression in root and nodule tissues. Polyamines are well established as regulators of nodulation, influencing nodule number, biomass, and rhizobial attachment. Optimal concentrations of polyamines promote nodulation, whereas excess levels impair function (Terakado et al., 2006). In soybean, altered spermidine and spermine levels have been shown to affect nodule formation, as in the supernodulating mutant En6500, while uncommon polyamines and spermine analogs have been detected exclusively in nodules (Terakado et al., 2006). In *Lotus japonicus*, polyamine biosynthesis genes such as *LjSPDS* and *LjSPMS* are strongly expressed in cortical cells, vascular bundles, and central nodule tissues early in development, with systemic changes across plant organs (Efrose et al., 2008). Consistent with these findings, the expression patterns and functional annotation of *Glyma.18G137100* and *Glyma.08G303800* suggest that they may regulate not only nodule development and biomass but also spatial distribution, potentially influencing infection site selection through spermidine/spermine catabolism and H_2_O_2_-mediated infection thread formation.

Additionally, the functional roles of other genes located near significant SNPs, as well as those identified through interaction analysis, warrant further investigation (**Table 1**). Because the traits examined in this study are novel, functional characterization of the associated genes will be essential for providing insights into the mechanisms underlying these traits and for clarifying their contribution to nodulation dynamics.

## 5 Conclusion

This report describes the cultivation of diverse soybean accessions in a hydroponic system inoculated with *Bradyrhizobium* bacteria to promote nodule formation. The seedlings were initially germinated for seven days on brown germination paper before being transferred to the hydroponic setup. The innovation in this study lies in the development of an affordable, end-to-end phenotyping system for these plants, integrating deep learning-based batch image pre-processing and root nodule feature extraction. We developed a phenotyping pipeline that combines image processing and analysis of plant roots with nodules, using Smart Shooter imaging software to capture high-quality images. This platform offers a high-throughput, cost-effective solution capable of generating biologically relevant time-series data on a wide range of nodulation traits. The integration of both hardware and software components provided a reliable, efficient, and repeatable root nodule phenotyping platform. Additionally, we demonstrated the precision of our pipeline by comparing it with manually measured nodule traits, showing high accuracy and efficiency. Since QR code labeling is widely used for tracking plant accession and replication information, we validated that our platform does not misidentify QR code dots as nodules.

We further demonstrated the downstream application of this pipeline by conducting GWAS using two widely accepted approach like FarmCPU. Using phenotyping data for 24 nodulation traits obtained through our platform, along with genotypic data from the soybean accessions, we identified 29 unique significant SNPs associated with the traits. Furthermore, using annotation data, publicly available expression profiles, and interaction analysis, we identified putative candidate genes located within 50 kb upstream and downstream of the significant SNPs. Future studies are needed to validate these candidate genes as molecular targets in plant transformation experiments to evaluate their impact on nodulation efficiency in soybean. Additionally, the significant SNPs identified in this analysis will serve as valuable resources for future soybean breeding programs, particularly those focused on developing markers for root nodulation traits. Collectively, our findings provide a foundation for uncovering the genetic components underlying phenotypic variation in root and nodulation traits in soybean landraces.

Future studies are necessary to extend this newly developed software pipeline to other important nodulating plant species such as *Phaseolus vulgaris* (common bean), *Vigna unguiculata* (cowpea), *Medicago sativa* (alfalfa), and *Vicia faba* (faba bean). Adapting this platform for these species would enable high-throughput phenotyping of a wide range of nodulation traits and facilitate genomic prediction and GWAS to identify genomic regions associated with nodulation. The utilization of the identified candidate genes and SNPs in future breeding programs aimed at enhancing biological nitrogen fixation could significantly reduce the reliance of modern agriculture on commercial nitrogen fertilizers.

## Data Availability

The pipeline developed in the study is publicly available in a GitHub repository at: https://github.com/Salk-HarnessingPlants-Initiative/soybean-nodule-detection. All original contributions are included within the article and its **Supplementary Materials**. For additional information or inquiries, readers are encouraged to contact the corresponding authors.

## Supplementary material

Supplementary Figure 1 Representative root systems showing variation in nodule number among soybean accessions. **(a)** Plant with fewer nodules, exhibiting a smaller nodulation zone along the primary root. **(b, c)** Plants with greater nodule numbers, displaying broader nodulation zones and greater spatial distribution of nodules along the primary root.

Supplementary Figure 2 Representative output image demonstrating accurate localization of nodules within a complex root background, with a nodule count exceeding 70.
