# Supplementary figures and images for: YOLO-based high-throughput phenotyping pipeline for soybean nodulation traits in genomic research
Source: Front Plant Sci. 2026 Apr 21;17:1816132. doi: 10.3389/fpls.2026.1816132 (PMC13139125; doi:10.3389/fpls.2026.1816132)

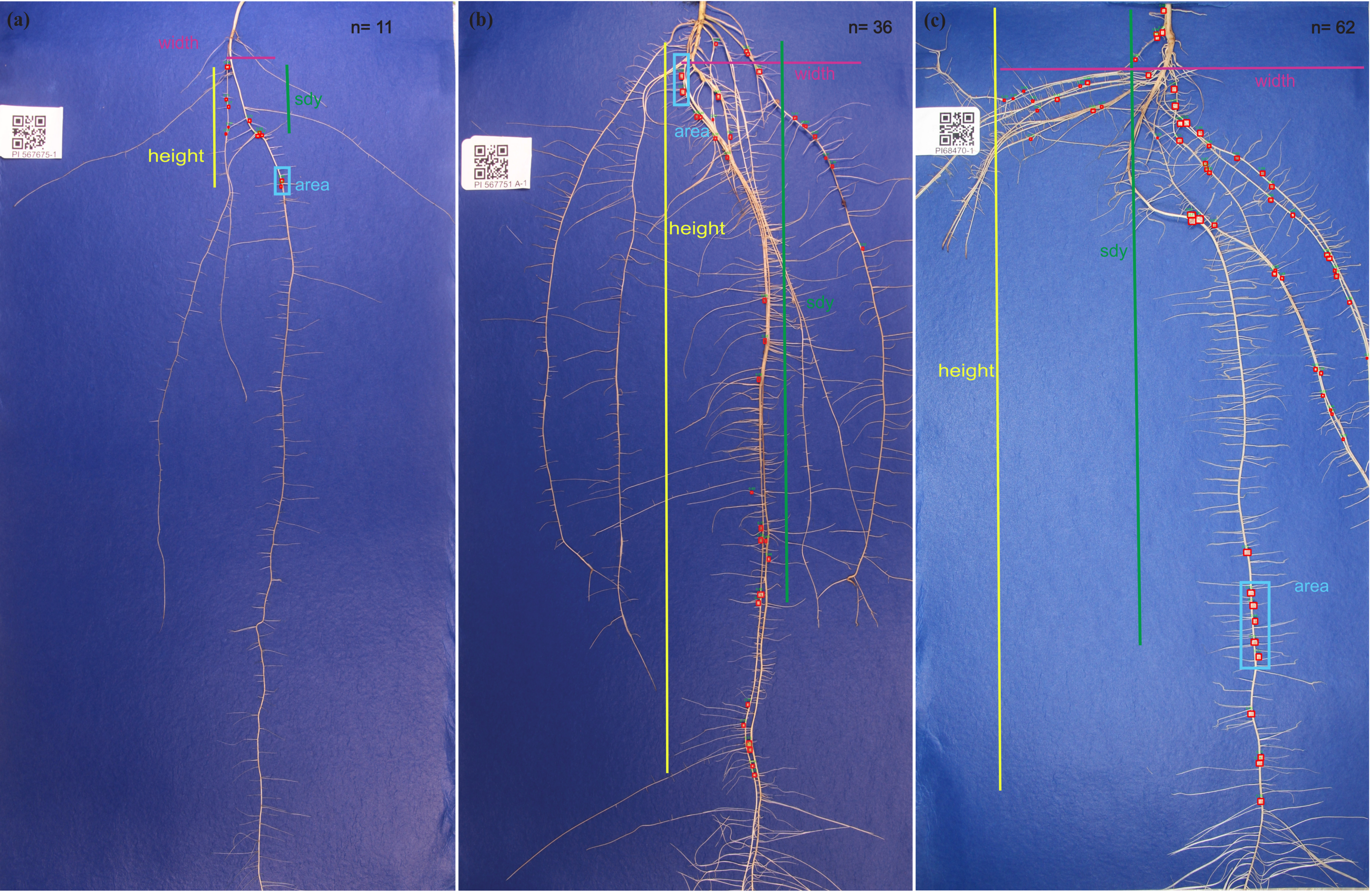

Supplement: Supplementary Figure 1 — Representative root systems showing variation in nodule number among soybean accessions. (a) Plant with fewer nodules, exhibiting a smaller nodulation zone along the primary root. (b, c) Plants with greater nodule numbers, displaying broader nodulation zones and greater spatial distribution of nodules along the primary root. [file Image1.jpeg]

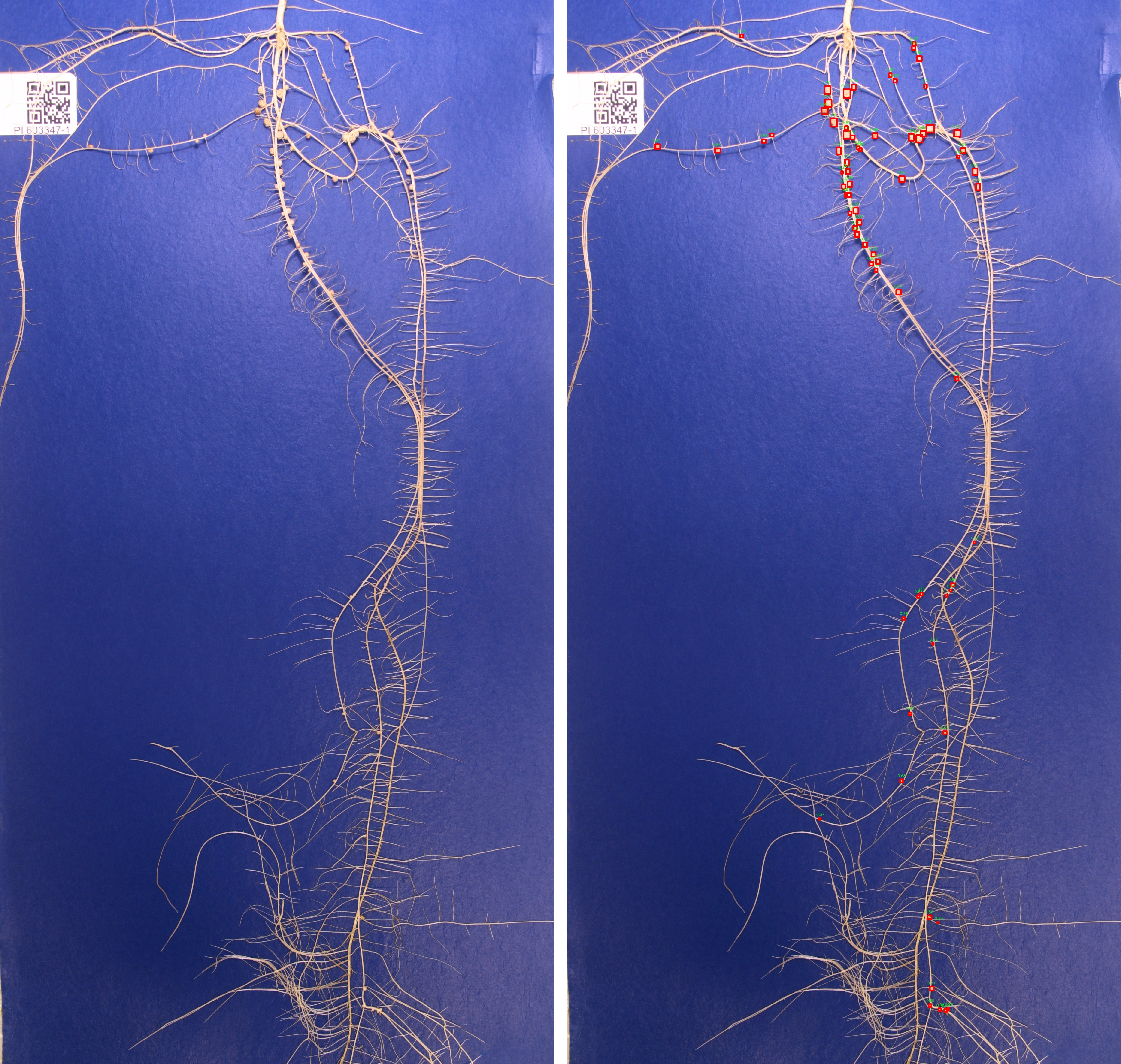

Supplement: Supplementary Figure 2 — Representative output image demonstrating accurate localization of nodules within a complex root background, with a nodule count exceeding 70. [file Image2.jpeg]
